# The influence of post-glacial migration and hybridization on the gene pool of marginal *Quercus pubescens* populations in Central Europe

**DOI:** 10.1093/aob/mcae216

**Published:** 2024-12-19

**Authors:** Jil Pütz, Simon Jansen, Oliver Reutimann, Christian Rellstab, Sándor Bordács, Charalambos Neophytou

**Affiliations:** Department of Forest and Soil Sciences, Institute of Silviculture, BOKU University, Peter-Jordan-Str. 82, AT-1190 Vienna, Austria; Department of Forest and Soil Sciences, Institute of Silviculture, BOKU University, Peter-Jordan-Str. 82, AT-1190 Vienna, Austria; Institute of Integrative Biology, ETH Zurich, CH-8092 Zurich, Switzerland; Swiss Federal Research Institute WSL, CH-8903 Birmensdorf, Switzerland; Department of Botany, Hungarian University of Agriculture and Life Sciences, Ménesi út 42-44. HU-1118 Budapest, Hungary; Department of Forest and Soil Sciences, Institute of Silviculture, BOKU University, Peter-Jordan-Str. 82, AT-1190 Vienna, Austria; Department of Forest Nature Conservation, Forest Research Institute of Baden-Württemberg (FVA), Wonnhaldestr. 4, D-79100 Freiburg, Germany

**Keywords:** *Quercus pubescens*, downy oak, genetic diversity, genetic differentiation, haplotype, hybridization, introgression, peripheral populations, white oaks, microsatellites, short sequence repeats

## Abstract

**Background and Aims:**

In Central Europe, the drought-tolerant downy oak (*Quercus pubescens*) is at the northern edge of its natural distribution range, often growing in small and spatially isolated populations. Here, we elucidate how the population genetic structure of Central European *Q. pubescens* was shaped by geographical barriers, genetic drift and introgression with the closely related sessile oak (*Q. petraea*).

**Methods:**

In total, 27 *Q. pubescens* populations from the northern margin of its natural distribution range were sampled. Based on 16 nuclear microsatellite markers (nSSRs), Bayesian clustering and distance-based analyses were performed to determine the intraspecific genetic structure and to identify genetic barriers. To identify drivers of introgression with *Q. petraea*, generalized linear models were applied to link levels of introgression with environmental conditions. To track post-glacial migration routes, the spatial distribution of haplotypes based on eight chloroplast microsatellite markers (cpSSRs) was investigated.

**Key Results:**

Based on nSSRs, the study populations of *Q. pubescens* were divided into a western and an eastern genetic cluster. Within these clusters, more pronounced genetic substructure was observed in the west, probably due to a rugged topography and limited gene flow. Introgression from *Q. petraea* was more prevalent at wetter and north-exposed sites and in the west. The identified cpSSR haplotypes followed known migration pathways.

**Conclusions:**

Our results suggest two late-glacial refugia in or near the southwestern Alps and the southeastern Alps as potential sources for post-glacial migration. Although some genetic exchange is evident in northern Italy, south of the Alps, the two clusters remain distinct at a large scale. Landscape features and introgression with *Q. petraea* shaped the genetic substructure at a smaller scale. Our study provides a comprehensive overview of the genetic structure of *Q. pubescens* in Central Europe, relevant for conservation.

## INTRODUCTION

The spatial pattern of population genetic structure and diversity is the result of past evolutionary processes, including genetic drift, gene flow and natural selection. However, the way these processes shape the spatial distribution of genetic variation in a species’ range remain a topic of ongoing scientific debate. For instance, a widely accepted theory based on the central-abundance hypothesis (Brown, 1984), which claims that genetic diversity is expected to be higher in populations situated at the centre of a species’ range and to decrease towards the periphery, has been challenged by several studies (Sagarin *et al.*, 2006; Samis and Eckert, 2007; Dauphin *et al.*, 2020; Ntuli *et al.*, 2020).

Given that genetic diversity underpins a species’ capacity to adapt, thrive and maintain resilience (e.g. Stange *et al.*, 2021), it is essential to gain a thorough understanding of how evolutionary mechanisms shape genetic variation (Eckert *et al.*, 2008). Species-specific multi-population studies may therefore assist in (1) identifying the specific drivers underlying the spatial distribution of genetic diversity, (2) developing more accurate models for the spatial distribution of population genetic diversity, or (3) evaluating the adaptive potential of species in the context of climate change. In light of ongoing climate change and the associated shifts in species’ distribution ranges, it is becoming increasingly important to gain insights into the genetic constitution of peripheral populations, particularly in terms of their role in colonizing new habitats (e.g. Roger *et al.*, 2012).

In Central Europe, the native white oak species (section *Quercus*; Hipp *et al.*, 2020) are expected to be more resilient to climate change impacts compared to most other native tree species and may therefore increase their importance and distribution range in the future (Mauri *et al.*, 2023; Kašpar *et al.*, 2024). Although each species has distinctive ecological requirements, there are several areas in Europe where their distribution ranges overlap, and where they can be found in sympatry. While *Quercus robur* (pedunculate oak) and *Q. petraea* (sessile oak) are widespread across large parts of Europe with temperate climate, *Q. pubescens* (downy oak) has its distribution range further south in sub-mediterranean deciduous forest communities, becoming more fragmented towards the north (Leuschner and Ellenberg, 2017). However, with ongoing global warming, *Q. pubescens* may (1) naturally expand its northern distribution, (2) become an economically important tree species due to its adaptation to relatively hot and arid conditions, and (3) serve as a reservoir of drought-resistant gene variants for other white oak species.

For tree species, which are long-lived and can only migrate through seed dispersal or pollen flow, peripheral populations may differ strongly in their demographic history and genetic composition among themselves or from the central populations (e.g. Fady *et al.*, 2016; Silvestro *et al.*, 2023). During the last glacial period, many tree species, including European white oaks, survived in spatially restricted refugia (Birks and Willis, 2008). For central European white oak populations, an origin from refugia located in the Mediterranean area is widely accepted (Brewer *et al.*, 2002). Towards the end of the last glacial period, warmer climatic conditions facilitated the migration of white oak species across Europe. In European white oaks, haplotypes of the maternally inherited chloroplast DNA (cpDNA) greatly reflect the refugial origin and routes of late-glacial and post-glacial migration (Petit *et al.*, 2002*a*). An Iberian cpDNA lineage is widespread in the west, mirroring a north–northeastward colonization out of the Iberian Peninsula. A Balkan lineage reflects an east-to-west migration route through Central Europe. An Italian (Apennine) lineage is roughly distributed along a south-to-north axis stretching from Italy over the Alps all the way to the Scandinavian Peninsula (Brewer *et al.*, 2002; Petit *et al.*, 2002*a*). Since this post-glacial range expansion, refugial populations came into recent secondary contact in Central Europe, leading to extensive gene flow among lineages and species over many generations (Petit *et al.*, 2003; Neophytou and Michiels, 2013).

In oaks, hybridization has been described for several closely related species, such as between *Q. pubescens* and *Q. petraea*, and the production of fertile progeny has been documented in many studies (e.g. Valbuena-Carabaña *et al.*, 2005; Salvini *et al.*, 2009; Rellstab *et al.*, 2016*a*; Reutimann *et al.*, 2020). Recurrent hybridization, backcrossing and introgression events have blurred species-specific haplotype patterns for recolonizing white oak species in central Europe (Petit *et al.*, 2002*b*). Furthermore, frequent hybridization between European white oak species has been discussed as an evolutionarily relevant mechanism in terms of adaptation and migration (Petit *et al.*, 2003; Leroy *et al.*, 2020). Northward and interspecific gene flow from Mediterranean oak populations has been reported as a relevant source of genetic diversity, representing a potential ‘migration route’ of pre-adapted gene variants during climate change (López De Heredia *et al.*, 2017). Additionally, introgression may facilitate spread into habitats outside the ecological optimum of a pure species (Leroy *et al.*, 2020; Wong *et al.*, 2022).

Today, *Q. pubescens* occurs naturally in large parts of Southern Europe. Its distribution range exhibits two centres: one including the areas around the Pyrenees, Southern France and most of the Italian peninsula and another, larger one, that includes the Balkan Peninsula and western Anatolia and is flanked by the Pannonian Basin to the north. These two centres are connected by a narrow strip south of the Alps, where *Q. pubescens* is naturally occurring. On the northern side of the Alps, the species is present in more or less spatially isolated populations in parts of Switzerland, eastern France and southwestern Germany. *Quercus pubescens* is completely absent in the area between southwestern Germany and a line between Czechia and eastern Austria. To the northeast of the Alps, natural populations can be found in areas within and around the Pannonian Basin (Caudullo *et al.*, 2024). Populations of *Q. pubescens* north of the Alps are presumed to be remnants of a wider distribution during the Holocene climatic optimum which was limited by climate cooling and the expansion of other, more competitive species such as *Fagus sylvatica* (common beech) since the Bronze Age (around 4200 years ago; Leuschner and Ellenberg, 2017; Reif and Trué, 2023).

In Central Europe, *Q. pubescens* is one of the most xerophilous tree species, generally found at altitudes between 200 and 1500 m above sea level and mainly occurring in habitats with steep slopes, strong sun exposure, rainfall <400 mm during the growing season, and winter temperatures between 0 and 5 °C (Bordács *et al.*, 2019). Although the species mainly occurs on calcareous substrates and is competitive on extremely dry sites (Rellstab *et al*., 2016b), it can grow on a wide range of soils, including crystalline bedrock and rocky sites. Due to its slow growth, high light requirements and limited growth capacity (maximum height of 20 m) the species is restricted to sites with low competition, mostly found in unproductive central European forest stands (Schütt, 1998; Sayer, 2000; Wellstein and Spada, 2015; Neophytou *et al.*, 2024). Even with its relatively low rate of exploitation compared to *Q. robur*, the species underwent significant population fragmentation due to land conversion, particularly for wine production or fruit orchards (Bordács *et al.*, 2019).

Overall, the species exhibits a high degree of morphological plasticity, leading to ongoing discussions about its taxonomic classification. Several subspecies and ecotypes of *Q. pubescens s.l.* have been described, such as *Q. virgiliana* and *Q. dalechampii* (Wellstein and Spada, 2015; Di Pietro *et al.*, 2020). However, intraspecific genetic studies using molecular markers [e.g. simple sequence repeats (SSRs)] have not yet been able to distinguish between the described morphotypes (Fortini *et al.*, 2022).

In this study, we focused on *Q. pubescens* populations from the northern edge of the species’ distribution range, within and around the Alpine mountain range. These populations represent the genetic source for natural northward migration in a warming climate and therefore will play a key role in future changes of species composition in temperate forests. Despite notable advances in understanding the genetic patterns in European white oaks (Milesi *et al.*, 2024; Degen *et al.*, 2021, 2022), comprehensive data on *Q. pubescens* remain scarce compared to other white oak species. Among others, earlier molecular genetic studies examined historical species distribution ranges and recolonization pathways via maternally inherited cpDNA markers (Petit *et al.*, 2002*c*; Slade *et al.*, 2008), or were focused on hybridization using nuclear SSRs (e.g. Salvini *et al.*, 2009; Chybicki *et al.*, 2012; Antonecchia *et al.*, 2015). However, previous studies were often geographically restricted or limited in the number of *Q. pubescens* populations (Neophytou *et al.*, 2015, 2024; Di Pietro *et al.*, 2020, 2021). Here, we provide a large-scale study with *Q. pubescens* populations from the peri- and inner-Alpine region, including previously unassessed study areas. By combining cpDNA and nuclear DNA markers, we consider fine-scale genetic structure, as well as post-glacial migration and recent gene flow, at both the inter- and intraspecific level. Due to their fragmented distribution, these peripheral populations provide an ideal case for studying the effects of demographic history on their spatial genetic structure and connectivity, and for comparing it with population genetic assumptions within a species’ margin or areas of convergence of post-glacial migration routes (Petit *et al.*, 2003).

Our primary objective was to assess the genetic diversity and differentiation of *Q. pubescens* populations in Central Europe. Additionally, we sought to determine the impact of landscape barriers and fragmentation on local population structure. We anticipated that the isolation and marginal character of our study populations, coupled with significant geographical barriers limiting gene flow, have resulted in a structured gene pool.

The second objective was to investigate the origin of *Q. pubescens* populations in Central Europe. Using maternally inherited cpDNA, we aimed to assign our populations to known post-glacial migration routes and determine whether this pattern was shaped by natural expansion or has been influenced by human-mediated seed transfer.

Finally, we quantified the extent of introgression between *Q. pubescens* and *Q. petraea* and linked it to site conditions. We hypothesized that introgression increases under conditions favourable for *Q. petraea*, such as higher precipitation. Conversely, less introgression is expected in warmer, drier climates on south-exposed terrain and/or soils with low water retention capacity.

## MATERIALS AND METHODS

### Study design

**Fig. 1.**
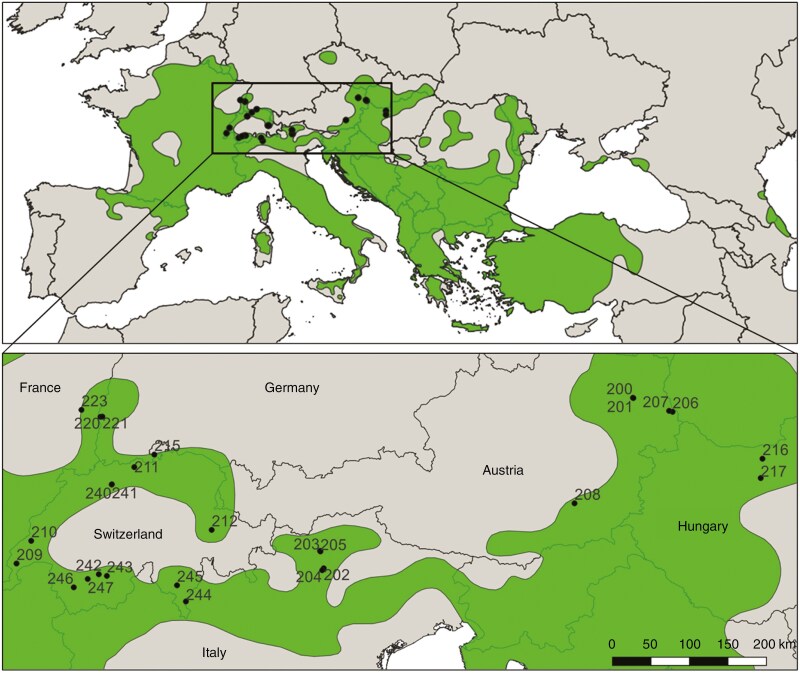
Study area and examined populations. The natural distribution range (Caudullo *et al.*, 2024) of *Quercus pubescens* is illustrated in green. The locations of the studied downy oak populations are shown with black dots. Population IDs are indicated in the enlarged map.

Overall, our study comprises 27 populations of *Quercus pubescens* located at the northern margin of the species’ natural distribution range including populations from Austria, France, Germany, Hungary, Italy and Switzerland (Fig. 1). The majority of populations are located in the areas surrounding the western part of the Alps, while seven are located in Central European areas east of the Alps (Fig. 1). Nineteen of them belong to the ongoing project ACORN (Jansen *et al.*, 2023). Five populations originate from Rellstab *et al.* (2016*a*, *b*) and Reutimann *et al.* (2020). The remaining three populations (208, 216, 217) were exclusively sampled for this study. The number of individuals sampled per population ranges from 17 to 47 (for population overview see Supplementary Data Table S1). For the ACORN populations and populations 208, 216 and 217, we sampled mature trees (diameter at breast height [1.3 m] ≥ 7 cm) and kept a distance of at least 25 m between them, to minimize a potential bias by family structures within populations. All sampled individuals were vital and showed a dominant or codominant growth within the stands. Each sampled tree was georeferenced, and its height and diameter at breast height were measured. For the samples of Rellstab *et al.* (2016*a*) and Reutimann *et al.* (2020), a minimum distance of 20 m was kept between individuals.

### Nuclear and chloroplast microsatellite markers

DNA extraction for populations 208, 216 and 217 and the ACORN populations was performed using buds or fresh leaves depending on the sampling period (spring/autumn) with a commercial kit (DNeasy® Plant Mini Kit, Qiagen) following the manufacturer’s protocol. DNA extraction of the other populations was performed as described in Rellstab *et al.* (2016*a*) and Reutimann *et al.* (2020) using the sbeadex maxi plant kit optimized for oak tree leaves (LGC Genomics) on a KingFisher 96 system (Thermo Scientific). Afterwards, we amplified 16 nuclear and eight chloroplast microsatellite markers with a PCR in three multiplex reactions. Three reference individuals were added to every plate as a standard. All chloroplast SSR (cpSSR, chloroplast microsatellite) markers were multiplexed in a single PCR: ccmp2, ccmp6 (Weising and Gardner, 1999), μcd4, μcd5, μdt1, μdt3, μdt4 and μkk4 (Deguilloux *et al.*, 2003). For the first multiplex (cpSSR), the PCR programme included an initial denaturation at 95 °C for 5 min, followed by 29 cycles with denaturation at 95 °C for 30 s, primer annealing at 52 °C for 90 s and elongation at 72 °C for 60 s and ending with a final elongation at 60 °C for 30 min. The nuclear SSR (nSSR, nuclear microsatellite) markers were amplified using two different multiplex reactions: FIR039, FIR053, GOT021, PIE099, PIE215, PIE227, WAG065 (Durand *et al.*, 2010), QMC00963 (Ueno *et al.*, 2008), QpZAG9, QpZAG15, QpZAG36, QpZAG104 (Steinkellner *et al.*, 1997), QrZAG11, QrZAG96, QrZAG112 (Kampfer *et al.*, 1998) and Quru-GA-1F02 (Aldrich *et al.*, 2002) (Supplementary Data Tables S2 and S3). For both nSSR multiplexes, a touchdown PCR was employed with an initial denaturation at 95 °C for 5 min, 10 cycles with denaturation at 95 °C for 30 s, primer annealing at 58 °C for 90 s and elongation at 72 °C for 30 s, a further 25 cycles with denaturation at 95 °C for 30 s, annealing for 60 s with temperature gradually decreasing from 58 to 55 °C and elongation at 72 °C for 30 s, as well as final elongation at 60 °C for 30 min. All individuals were genotyped with nSSRs, whereas cpSSRs were amplified for every second individual. To determine the length of the amplified fragments (alleles) in base pairs (bp), capillary electrophoresis was performed using the sequencer SeqStudio Genetic Analyzer from Applied Biosystems by Thermo Fisher Scientific. The software GeneMapper® Software 6 (Applied Biosystems) was used to perform peak scoring.

### Species identification

White oak species generally possess a high degree of phenotypic plasticity (e.g. Viscosi, 2015), frequently occur in sympatry and show substantial interspecific gene flow (Petit *et al.*, 2003; Salvini *et al.*, 2009), which can complicate species identification based on morphological traits in the field (Curtu *et al.*, 2007). Consequently, to identify the taxonomic identity and assess admixture for each study population and each individual, we extended our data set by each four reference populations of the closely related and sympatric species *Q. robur* and *Q. petraea* (Supplementary Data Table S1). All reference populations were selected from the ACORN project based on their geographical location, being (1) close to *Q. pubescens* populations and (2) equally representing the western and eastern parts of the study area. We used (1) the individual-based Bayesian genetic assignment method implemented in *Structure* 2.3.4, which attempts to delimit genetic clusters within which Hardy–Weinberg and linkage equilibrium hold (Pritchard *et al.*, 2000). To run *Structure* in parallel with 40 central processing units, we used *Strauto*, a Python script from Chhatre and Emerson (2017). We used *Structure* by conducting 20 independent runs for consecutive predefined numbers of assumed subpopulations (*K*) ranging from 1 to 10, applying 50 000 burn-in replications followed by 100 000 Markov chain Monte Carlo (MCMC) iterations. Correlated allele frequencies and the admixture model were chosen. Next, *Clumpak* (Clustering Markov Packager Across K) by Kopelman *et al.* (2015) was used to group, average and visualize the output from each K, using the *CLUMPP* method of Jakobsson and Rosenberg (2007). It was also used to identify different modes of clustering and average the mean ln probability of data, ln*P*(*X*|*K*) and its standard deviation per mode within a *K*. *Clumpak* was run using the online platform *StructureSelector* (Li and Liu, 2018) which we also used to calculate the metrics *MedMed K*, *MedMean K*, *MaxMed K* and *MaxMean K* according to Puechmaille (2016) (for more details, see below). For the interspecific *Structure* run, we selected the lowest *K* that differentiated all three species. We refrained from applying a formal method to select the number of *K* clusters (e.g. Evanno *et al.*, 2005; Puechmaille, 2016) as our goal was to identify the species and quantify admixture between species in a parsimonious way. To adjust the graphical output of *Structure* runs we used the program *Distruct* 1.1 (Rosenberg, 2004).

Subsequently, we classified individuals as pure or admixed based on their membership proportions to species-specific clusters. If an individual had a membership proportion (*q*-value) to a species-specific cluster greater than 0.875 it was assigned as *pure*, if the *q*-value was between 0.625 and 0.875 it was classified as *admixed* and if it was below 0.625 it was characterized as *highly admixed*. If more than one cluster appeared within a species, we added the membership proportions to each of these within-species clusters and performed the aforementioned classification with the sum of *q*-values of all intraspecific clusters. The rationale behind these thresholds is that pure trees, backcrosses and first-generation hybrids are expected to have a membership proportion of 1.0, 0.75 and 0.5 to a species cluster, respectively, so that these thresholds define intervals of membership proportions around these values. These have been proposed by simulation-based studies in oaks (Guichoux *et al.*, 2013; Neophytou, 2013). However, in natural populations, we expect to have more complex situations with advanced generation hybrids and backcrosses. Therefore, we kept the thresholds as described above, but decided to change the name of each category (*pure*, *admixed* and *highly admixed* instead of pure, backcrosses and first-generation hybrids).

In addition to Bayesian clustering, we applied a distance-based method to explore the relationships between *Q. pubescens* and the other two oak species. By using a reduced dataset with all individuals classified as *pure* for any of the three species, we calculated pairwise *F*_ST_ values according to Nei (1987) with the R-package *HIERFSTAT* (Goudet, 2005). Based on the pairwise distance matrix, we conducted a principal coordinates analysis (PCoA) with GenAlEx (Peakall and Smouse, 2006, 2012).

### Intraspecific genetic differentiation

To determine the intraspecific spatial genetic structure in *Q. pubescens*, we subsampled our initial data set to include only those individuals of each population assigned as *pure Q. pubescens*. *Structure* was used with the same settings as described above, except that we applied the *locprior* option (using population ID as location information), which is recommended when there is low genetic differentiation between the populations (Hubisz *et al.*, 2009). Since we wanted to detect structure at different hierarchical levels, we followed a different approach to identify the optimal number of *K* clusters. In particular, we considered the following criteria. First, we addressed whether additional meaningful substructure appeared with increasing *K*. For this purpose, we calculated the metrics *MedMed K*, *MedMean K*, *MaxMed K* and *MaxMean K* according to Puechmaille (2016) using *StructureSelector*. For each *K*, these metrics express the number of clusters to which at least one of our study populations was assigned with a *q*-value of ≥0.5. The metrics *MedMed K* and *MedMean K* express this number as the median across the 20 replicate runs performed for the given *K*. *MaxMed K* and *MaxMean K* express this number as the maximum across these runs for the given *K*. We rejected all *K* values that did not result in an increase of any of these statistics (criterion 1). Second, we addressed whether multimodality occurred among replicate runs for each *K* by comparing the modes inferred by *Clumpak*. We rejected *K* values with conflicting clustering solutions among modes (criterion 2). We selected the highest possible *K* fulfilling both criteria 1 and 2. Additionally, we observed the mean ln probability of data for each *K*-value – ln*P*(*X*|*K*) – and its standard deviation as an indication of hierarchical structure. If population structure is present, ln*P*(*X*|*K*) usually rises sharply for lower *K*-values and then plateaus with increasing *K*. The lowest value of *K*, after a plateau of ln*P*(*X*|*K*), has been reached, often expresses the uppermost level of hierarchical structure (Evanno *et al.*, 2005; Pritchard *et al.*, 2010; Grant and Chenoweth, 2021). After carrying out a *Structure* analysis across all *Q. pubescens*, we repeated the procedure by subdividing the populations into regional clusters as revealed by the intraspecific analysis with all populations until no meaningful clustering was obtained. This resulted in a hierarchical analysis which we repeated in lower-level regional sets of populations until no residual structure was evident.

As an alternative method to visualize patterns of intraspecific genetic structure in *Q. pubescens*, we conducted PCoA with GenAlEx (Peakall and Smouse, 2006, 2012) using pairwise *F*_ST_ values according to Nei (1987) calculated with the R-package *HIERFSTAT* (Goudet, 2005).

### Genetic diversity

To quantify genetic diversity, observed (*H*_o_) and expected heterozygosity (*H*_e_) were calculated using GenAlEx (Peakall and Smouse, 2006, 2012). The function *basic.stats* from the R package *HIERFSTAT* (Goudet, 2005) was applied to compute the inbreeding coefficient (*F*_IS_) for each population. The software ADZE (Szpiech *et al.*, 2008) was used to calculate allelic richness (AR) with rarefaction (Petit *et al.*, 1998). We performed analysis across all populations of *Q. pubescens*, and within genetic clusters inferred by the *Structure* analysis, including only individuals of each population which we previously classified as *pure Q. pubescens*. For comparison, we also quantified the genetic diversity in the *Q. petraea* and *Q. robur* reference populations by including individuals classified as *pure* to the corresponding species cluster.

### Geographical barriers and genetic differentiation

The software *Barrier v.2.2* (Manni *et al.*, 2004) was used to identify geographical barriers between populations which separate genetically differentiated populations. This software uses the Monmonier maximum difference algorithm (Monmonier, 1973) to identify regions with distinct genetic discontinuity. The coordinates from each population were mapped into a matrix linked by Delauney triangulation (Brassel and Reif, 1979). The barriers in the triangulation were identified with the help of one unbiased genetic distance matrix (Nei, 1987) and 100 bootstrapped unbiased genetic distance matrices (Nei, 1987), which were calculated with the software *MICROSAT* (Minch *et al.*, 1995). The optimal number of barriers was selected by minimizing the number of barriers and maximizing the bootstrap values (Neophytou *et al.*, 2011).

### Chloroplast haplotypic composition

For chloroplast haplotype nomenclature, we followed the studies of Neophytou and Michiels (2013) and Neophytou *et al.* (2024). Therefore, before naming the haplotypes, the allele sizes for each marker were adapted to Neophytou and Michiels (2013) by carrying out a ring test with samples from that study. After calling the haplotypes, the R package *POPPR* (Kamvar *et al.*, 2014) was used to define the phylogenetic relationships between them and create a minimum spanning network. Haplotypic diversity within population (*h*_S_) and differentiation among populations (*G*_ST_) were calculated using the software *PermutCpSSR* 2.0, which applies the formulas from Pons and Petit (1996).

### Genetic introgression and its correlation with environmental variables

To calculate the level of introgression (introgression index), the taxonomic assignments using *q*-values from the first, overall *Structure* analysis results were used. The introgression index can be expressed as (Neophytou *et al.*, 2024):


$$\begin{array}{l} IG_{nu}=\;\frac{n_{had}+0,5n_{ad}}{n} \end{array}$$

where *n*_had_ is the number of individuals that are *highly admixed* (*q*-value < 0.625), *n*_ad_ is the number of individuals that are *admixed* (*q*-value = 0.625–0.875) and *n* is the total number of individuals.

To link the introgression index with environmental conditions, environmental data were obtained from CHELSA 2.1 with a 30-arc-second resolution (Karger *et al.*, 2017, 2021). We used the 19 bioclimatic variables based on the reference period of 1981–2010, which represent climatic descriptors related to temperature (11 descriptors) and precipitation (eight descriptors), calculated from monthly means and maxima. Additionally, we used the digital elevation model GLO-30 provided by Copernicus with a resolution of 30 m for topographic data, from which we calculated variables such as slope, northness, eastness and topographic wetness index (TWI) (for variable overview, see Supplementary Data Table S4). For calculating TWI, we used the D8 method implemented in the Python package *Physheds* (Bartos, 2020). We used generalized linear models (GLMs) to link the introgression index of populations to the environmental and topographic variables, similar to that described in Reutimann *et al.* (2023). Pure *Q. robur* and *Q. petraea* individuals (*q*-value > 0.875) and populations 209–214 (due to the lack of individual coordinates) were excluded prior to the analysis and population-level values were derived by averaging individual-level data. To minimize multicollinearity among environmental descriptors, we excluded variables that were highly correlated with each other. Therefore, we conducted pairwise Pearson’s correlations among all environmental variables (Table S5) and implemented the following criteria for variable selection (similar to Leempoel *et al.*, 2015). First, derived variables showing high correlation (*r* ≥ |0.7|, Dormann *et al.*, 2013) with primary variables were excluded (with one exception: we retained wetness index over slope, as it was deemed biologically more relevant). Second, in cases where two primary variables exhibited high correlation (*r* ≥ |0.7|), we chose the biologically more relevant one. Before conducting the analyses, all selected variables were scaled (s.d. = 1) and centred (mean = 0).

Population-level GLMs consisted of the introgression index as response variable and mean environmental variables as explanatory variables. Models were fitted using the *gamlss* function of the *GAMLSS* R-package (Rigby and Stasinopoulos, 2005). We used a beta (logit-logit, BE) distribution family to model the response variable, which is limited between zero and one (Douma and Weedon, 2019). We then used the *stepGAIC* function of the *GAMLSS* R-package to perform stepwise model selection (both directions) based on the Akaike information criterion (AIC). After model selection, we tested for variance inflation (VIF) using the *vif* function of the *CAR* R-package. Model selection was repeated excluding the variable with the highest VIF from the full model until no variables exceeded a VIF value of 3 (Zuur *et al.*, 2010). We evaluated the final models based on generalized *R*^2^ values calculated using the *Rsq* function of the *GAMLSS* R-package.

## RESULTS

### Species identification

**Fig. 2.**
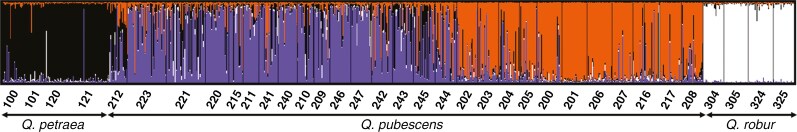
Species identification: *Structure* barplot with 27 putative *Q. pubescens* populations, four *Q. petraea* populations and four *Q. robur* populations (*K* = 4). Each box represents a study population, each bar within a box illustrates an individual while the inferred clusters are shown with different colours.

The first *Structure* analysis to identify the taxonomic identity of each individual included all 27 *Q. pubescens* and the four reference populations of *Q. petraea* and *Q. robur*, totalling 948 individuals (Fig. 2). For *K* = 3, *Structure* failed to identify the species as *Q. petraea* clustered together with part of the *Q. pubescens* populations, in particular those situated in the western part of our study area (Supplementary Data Fig. S1). The most congruent clustering result, based on *Clumpak*, was achieved with *K* = 4 (Fig. S1; Table S6), separating all reference populations according to their taxonomic identity and showing two genetic clusters within *Q. pubescens* (Fig. 2). This choice was also supported by the fact that *K* = 4 marked the start of a plateau of ln*P*(*X*|*K*) and the lack of multimodality meaning that all 20 replicate runs gave the same clustering solution (Table S6; Fig. S2). The metrics *MedMed K*, *MedMean K*, *MaxMed K* and *MaxMean K* (Puechmaille, 2016) suggested more subdivision into 6–8 clusters (Fig. S3), but clustering solutions (*K* = 6–8) were mostly not congruent (Fig. S4). In conclusion, and according to the aim to assign species identity and quantify species admixture, we chose *K* = 4 as the optimal number of clusters. Based on *Structure*, all putative *Q. pubescens* populations were correctly assigned, i.e. exhibited the highest membership proportion to the clusters corresponding to *Q. pubescens*, except population 212 (Chur, Switzerland), which displayed higher membership proportion to *Q. petraea* rather than to *Q. pubescens*.

The remaining *Q. pubescens* populations were divided into two different clusters (coloured orange and blue in Fig. 2). The first intraspecific cluster was more prevalent in the western part of our study area, including populations 244 and 245 in Ticino and all other populations to the west. The second intraspecific cluster included all other populations in the east, beginning from South Tyrol (populations 202–205; Figs 1 and 2). As population 212 only had one individual identified as pure *Q. pubescens*, with the rest being assigned as *Q. petraea* or *highly admixed*, it was removed from further analyses. Without the reference populations of *Q. petraea* and *Q. robur*, as well as population 212, the total number of individuals was reduced to 690, with 510 being identified as pure *Q. pubescens*, 13 as *Q. petraea* and two as *Q. robur* (*q* > 0.875). The remaining trees were classified as follows: 126 *admixed* individuals [0.875 > max(*q*) > 0.625] and 39 assigned as *highly admixed* based on their maximum *q*-value being below 0.625 (Supplementary Data Table S7).

**Fig. 3.**
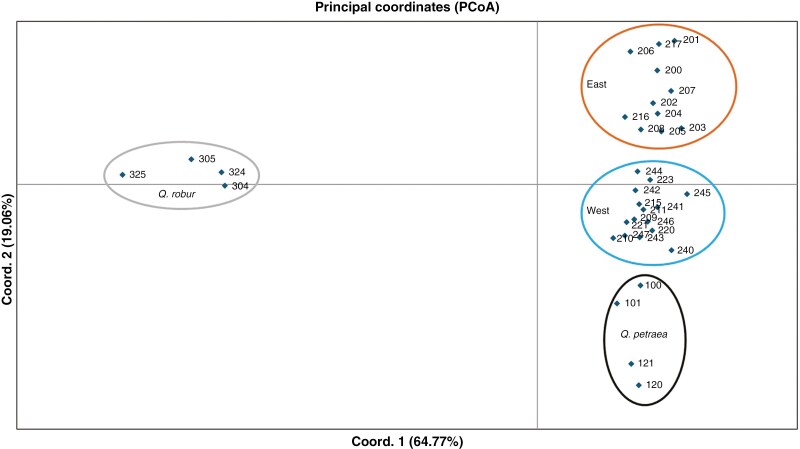
Principal coordinate analysis (PCoA) of all studied *Q. pubesce*ns populations (only pure individuals) and *Q. petraea* and *Q. robur* reference populations based on pairwise *F*_ST_ values after Nei (1987) (for values see Supplementary Data Table S8).

The subdivision into four clusters was also supported by the *F*_ST_-based PCoA which included all pure individuals of the three species. This analysis separated all three species and showed further subdivision within the *Q. pubescens* populations into a western and an eastern cluster in accordance with *Structure* analysis (Fig. 3).

### Intraspecific genetic differentiation

**Fig. 4.**
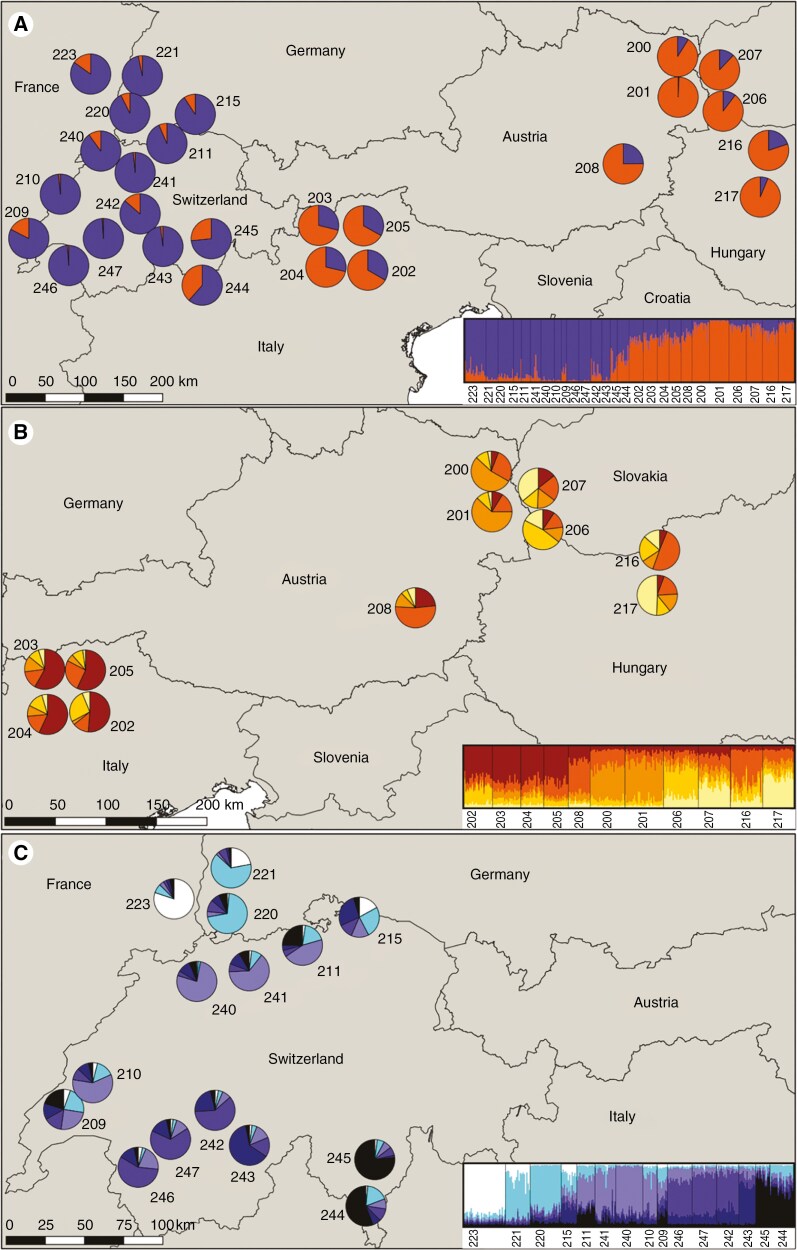
Population genetic structure of *Q. pubescens.* Each population is represented by a pie chart and the colours indicate the membership proportion for each genetic cluster inferred by *Structure*. (A) *K* = 2 for all pure *Q. pubescens*; (B) *K* = 5 for Eastern populations; (C) *K* = 6 for Western populations.

To address intraspecific genetic differentiation among *Q. pubescens* populations, we compiled a new data set, including the 510 individuals identified as pure in the initial *Structure* run. We identified *K* = 2 as the optimal number of clusters, because (1) it led to a sharp increase of ln*P*(*X*|*K*) which was not continued for *K* = 3 (Supplementary Data Fig. S5), (2) all runs for this *K* were unimodal with a high similarity score among runs (Table S9) and (3) it provided a meaningful result separating populations from the east and west (Fig. 4A; Fig. S6). Even if Puechmaille’s (2016) measures *MedMed K*, *MedMean K*, *MaxMed K* and *MaxMean K* suggested further subdivision, up to *K* = 8, we decided not to select a *K* higher than 2, because the different replicate runs within a given *K* delivered conflicting results (Table S9; Fig. S7). In particular, for *K* = 3–7 some runs detected more substructure in the west while others subdivided the eastern cluster (Figs S6 and S8). Even for *K* = 8, where no multimodality occurred, we observed substantial differences between the *q*-values among runs, which explains the high standard deviation of ln*P*(*X*|*K*) (Fig. S5) and the low similarity index among the runs (Table S9). Also, an intraspecific PCoA with pairwise *F*_ST_-values (Nei, 1987) confirmed the division into two clusters with the east and the west populations (Fig. S9).

Thus, following the hierarchical clustering approach, we created two new data sets, one containing 254 individuals from 15 western populations and the other containing 256 individuals from 11 eastern populations. Within the western cluster, we selected *K* = 6 as the optimal number of clusters of the *Structure* analysis. This solution explained a maximum of regional structure and was supported by each of Puechmaille’s (2016) metrics. Therefore, we selected the major mode for *K* = 6 as the most meaningful solution. Although bimodality was observed, we did not reject this *K* given the minor clustering differences between the modes (see Supplementary Data Figs S10–13; Table S10). We detected genetic clusters for the populations from Jura (210, 211, 240, 241; except 209 which was highly admixed), Valais (242, 246, 247; except 243 which was genetically distinct), Ticino (244, 245) and Kaiserstuhl (220, 221) (Fig. 4C). Population 223 is differentiated from the other western populations and forms its own cluster (Fig. 4C). No specific cluster was detected for populations 209 and 215 (Fig. 4C).

For the eastern cluster we chose *K* = 5 as the optimal number of clusters as suggested by the Puechmaille (2016) metrics and given that ln*P*(*X*|*K*) fell drastically after *K* = 5 while the results for this *K* were congruent (Supplementary Data Table S11; Figs S14–S17). Populations 200 and 201 from the Vienna Woods formed a cluster, whereas the four populations from South Tyrol (202–205) also formed a separate one (Fig. 4B). The subdivision of the remaining populations was less clear with high admixture. For each the three remaining clusters, at least one population was found with *q* > 0.5, but also a high degree of admixture was observed (Fig. 4B).

### Genetic diversity and differentiation

Based on the *Structure* results, we subdivided the populations of *Q. pubescens* into two groups, hereafter called *east* and *west* subsets. The *east* subset showed higher allelic richness, and higher observed and expected heterozygosity than the *west* subset (Table 1; Supplementary Data Table S12). The *Q. petraea* and *Q. robur* reference populations showed overall lower allelic richness and lower observed and expected heterozygosity than the *Q. pubescens* populations (Table 1). Within *Q. pubescens*, the inbreeding coefficient (*F*_IS_) was higher for the *west* subset. *F*_ST_ and Jost’s *D*_EST_ values were lower in the *east* than in the *west*, but overall, the *Q. pubescens* populations exhibited a higher *F*_ST_ and *D*_EST_ than the *Q. petraea* and *Q. robur* populations (Table 1).

**Table 1.** Genetic diversity and differentiation for pure *Quercus pubescens*, *Quercus petraea* and *Quercus robur*, as well as the subsets *east* and *west* for *Q. pubescens*.

Population	*N*_pop_	*N*_ind_	AR_8_	*H*_o_	*H*_e_	*F*_IS_	*F*_ST_	*D*_EST_
*Quercus pubescens*	25	502	5.926 ± 0.010	0.706 ± 0.011	0.708 ± 0.010	0.000 ± 0.015	0.058 ± 0.005	0.092 ± 0.028
Subset *east (Q.pubescens)*	11	256	6.069 ± 0.013	0.728 ± 0.016	0.707 ± 0.015	−0.029 ± 0.007	0.034 ± 0.002	0.041 ± 0.012
*Subset west (Q.pubescens)*	14	246	5.814 ± 0.014	0.689 ± 0.015	0.708 ± 0.013	0.022 ± 0.025	0.049 ± 0.003	0.054 ± 0.017
*Quercus petraea*	4	116	5.412 ± 0.016	0.683 ± 0.022	0.679 ± 0.022	−0.014 ± 0.015	0.029 ± 0.004	0.044 ± 0.016
*Quercus robur*	4	106	5.797 ± 0.024	0.696 ± 0.027	0.683 ± 0.025	−0.019 ± 0.009	0.029 ± 0.004	0.049 ± 0.015

*N*_pop_ = number of populations, *N*_ind_ = number of individuals, AR_8_ = allelic richness with rarefaction (rarefaction size = 8 individuals) ± s.e., *H*_o_ = observed heterozygosity ± s.e., *H*_e_ = expected heterozygosity ± s.e., *F*_IS_ = inbreeding coefficient ± s.e., *F*_ST_ = genetic differentiation ± s.e., Jost’s *D*_EST_ = genetic differentiation ± s.e.

### Geographical barriers and genetic differentiation

**Fig. 5.**
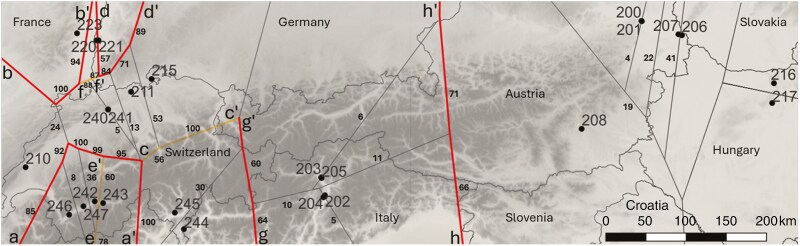
Gene flow barriers among *Q. pubescens* populations. The eight selected barriers (a–h) are shown with orange or red lines (different colours are used just for visualization). The beginning of a barrier is marked by the letters a–h and the end by aʹ–hʹ. Beside each segment of a barrier line is a number which indicates the bootstrap value.

*Barrier* identified eight gene flow barriers with bootstrap values ranging from 60 to 100 (Fig. 5). Barrier ‘a’ was the most prominent, separating the populations in Valais (242, 243, 246 and 247) from the rest. Barrier ‘c’, with bootstrap support of 56–100, indicated a genetic separation between 211 (Remigen, Aargau, Switzerland) and 215 (Osterfingen, Schaffhausen, Switzerland) on the one hand, and the populations from Ticino (244 and 245) on the other. The three Upper Rhine populations were all separated by barriers. Barrier ‘b’ isolated population 223 (Sigolsheim, Alsace, France), barrier ‘d’ isolated population 221 (Bitzenberg, Kaiserstuhl, Germany) and barrier ‘f’ isolated population 220 (Büchsenberg, Kaiserstuhl, Germany) from the remaining populations in the west. Despite having a relatively high bootstrap value of 53, no barriers were identified between 215 (Osterfingen, Schaffhausen, Switzerland) and the other Jura populations (i.e. 210, 211, 240 and 241). Barrier ‘e’ separated population 243 from the rest of the Valais group. Barriers ‘g’ and ‘h’ enclosed the South Tyrolean populations. No significant barrier was found for the remaining populations in the east.

### Chloroplast haplotypic composition

**Fig. 6.**
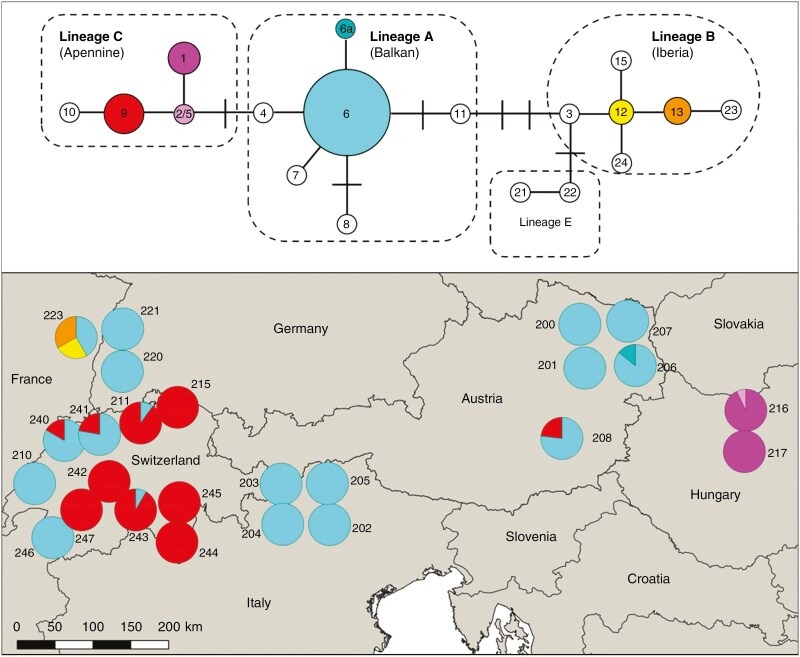
Minimum spanning network (at the top) of cpSSR haplotypes. Vertical lines represent missing haplotypes. Reference haplotypes from refugial areas (Neophytou and Michiels, 2013) were used to construct the minimum spanning network (colourless circles). Haplotypes found in this study are indicated with different colours. The size of the circle reflects the frequency of the haplotypes. Map (at the bottom) with pie charts showing the different haplotypes within different populations.

Within our studied populations, haplotype lineages A, B and C were represented, corresponding to three glacial refugia, Balkan, Iberia and Apennine, respectively (Petit *et al*., 2002c) (Fig. 6; Supplementary Data Fig. S13). A list of haplotypes and the correspondence between cpSSR and RFLP haplotypes are presented in Table S13. Haplotype 6 (Balkan) was the most abundant, representing over 60 % of the individuals, followed by haplotype 9 (Apennine) which was present in a quarter of the individuals. Haplotype 2/5 (Apennine) was only found once in population 216 in Hungary. Haplotype 1 (Apennine) could only be detected in the two Hungarian populations (216, 217). Haplotype 6a (Balkan) was also a rare haplotype that appeared in two individuals in population 206. Haplotypes 12 and 13 (Iberia) were only present in population 223. Haplotypic diversity within a population (*h*_S_) was low, both in the eastern and western population clusters (Table S14) with the differences between them being non-significant (Mann–Whitney test; *U* = 75, *P* = 0.657).

### Introgression and environmental variables

**Fig. 7.**
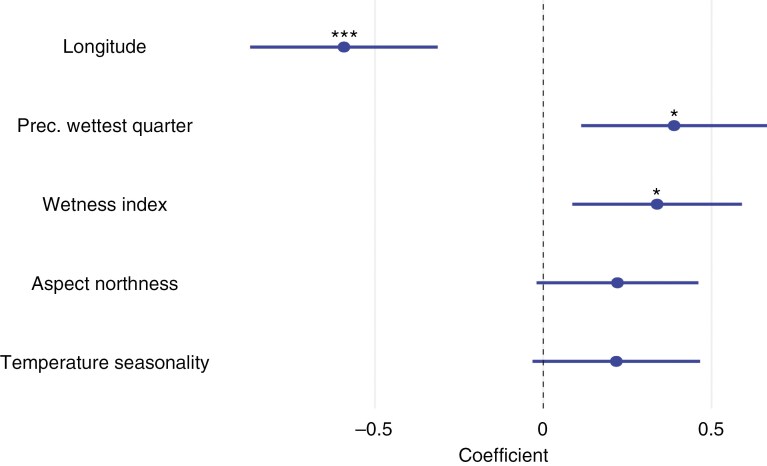
Effect of environmental descriptors on introgression index of *Q. pubescens* populations. Shown are the descriptors and their coefficients (error bars: 95% confidence intervals) included in the best model (generalized *R*^2^ = 0.59) resulting from the model selection procedure (*P < 0.05, **P < 0.01 and ***P < 0.001).

To investigate potential drivers of introgression in *Q. pubescens*, we associated environmental variables with the introgression index of populations through GLMs. After removing correlated environmental factors, 16 geographical, topographic and bioclimatic variables were included in the full model (Supplementary Data Table S5) and 14 variables remained after excluding variables with a VIF > 3. Stepwise model selection resulted in a model with a generalized *R*^2^ of 0.59, capturing the relationship between the introgression index and five environmental variables (Fig. 7; Table S15). Among the significant predictors (*P* < 0.05) longitude had the strongest (negative) effect on the introgression index (*P* < 0.001), followed by positive effects of precipitation of the wettest quarter (*P* < 0.05) and wetness index (*P* < 0.05). In addition, the final model included two non-significant terms: northness (*P* = 0.09, positive effect) and temperature seasonality (*P* = 0.11, positive effect).

## DISCUSSION

### Remarkable genetic differentiation between a western and an eastern genetic cluster may still carry the imprints of post-glacial migration

Our results show a rather distinct subdivision of *Q. pubescens* in our study area into a western and an eastern genetic cluster. Using nuclear microsatellites, we identified a pronounced genetic barrier situated between Ticino and South Tyrol, separating the two clusters with a zone of admixture between them. This subdivision was supported by the Bayesian clustering (*Structure*) analysis and the distance-based multivariate analysis. It is remarkable that the populations from Ticino clustered together with the other western populations in Switzerland, eastern France and southwestern Germany, although significant landscape barriers occur within this area, potentially preventing gene flow. Being situated south of the Alps, Ticino is separated from the populations in Valais to the west by high mountain ranges with their lowest point being the Simplon pass (2006 m a.s.l.), which is above the current maximum altitude of *Q. pubescens* natural occurrence in the area (1200 m; Brändli, 1996). Forests with *Q. pubescens* north and south of the main Alpine ridge (Alpine watershed) are isolated from each other by mountains, not only in the vicinity of Ticino, but also along most of the mountain range, except its southwestern tip at lower altitudes of the Maritime Alps, and the eastern foothills of the range in Eastern Austria (Pasta *et al.*, 2016). On the other hand, it is noteworthy that the populations from South Tyrol, also situated south of the main Alpine ridge, display a stronger genetic affinity to their counterparts in Austria and Hungary, rather than to Ticino and the western cluster. Geographically, South Tyrol is closer to most populations of the western cluster than to the Austrian and Hungarian populations. Considering the natural distribution of *Q. pubescens* (Fig. 1) and the distance of hundreds of kilometres along the southeastern margin of the Alpine range between the South Tyrolean populations and their Austrian and Hungarian counterparts, this relatedness is remarkable.

We interpret the subdivision into a western and an eastern cluster as the result of different postglacial migration histories and limited gene flow between the two genetic clusters for *Q. pubescens* in northern Italy. These migration events are mirrored by the analysis of chloroplast microsatellites. Notably, haplotype 6 is fixed for all South Tyrolean populations whereas haplotype 9 is fixed in Ticino. A common origin of all populations of the eastern genetic cluster, including South Tyrol, is likely, given their haplotypic composition. Secondary refugia were probably involved in early Holocene migration of *Q. pubescens* in this area. In particular, following an initial late glacial expansion, white oaks were able to survive the cold and dry Younger Dryas period (11 00010 000 years BP) forming such secondary refugia in the southeastern Alps and around the Pannonian basin (Bordács *et al.*, 2002; Slade *et al.*, 2008). These served as the main source of early post-glacial migration of white oaks in Central Europe (Brewer *et al.*, 2002). Our results from both chloroplast and nuclear markers agree with the hypothesis that *Q. pubescens* populations of the eastern cluster, including the South Tyrolean populations, originated from this refugium.

Conversely, populations of the western cluster must have primarily originated from the south and southwest. The occurrence of haplotype 9 of the Apennine lineage can be attributed to a northward migration crossing the Alps at the Simplon pass and advancing toward the Jura mountains (Mátyás and Sperisen, 2001; Csaikl *et al.*, 2002). Refugial populations in the northern Apennines and southwestern Alps might have given rise to this recolonization pathway. It has been suggested that *Q. pubescens* was involved in this migration across the Alps taking place during the climatic optimum in the Atlantic period (Csaikl *et al.*, 2002; Petit *et al.*, 2002*a*). Further north, the presence of haplotype 6 rather reflects the early Holocene (prior to the Atlantic period) east-to-west migration, originating from secondary refugia in the southeastern Alps and around the Pannonian Basin, which might have preceded the south-to-north colonization across the Alps (Brewer *et al.*, 2002; Petit *et al.*, 2002*a*). It has been hypothesized that *Q. petraea* was the species involved in this westward migration (Petit *et al.*, 2002*a*). We suggest that the presence of this haplotype in *Q. pubescens* is the result of introgression with *Q. pubescens* successfully pollinating the related *Q. petraea* and perhaps also *Q. robur* (to a lesser extent), as well as their hybrids. This is likely because *Q. pubescens* is a particularly successful pollinator when interbreeding with these species (Lepais and Gerber, 2011). In this way, a successful pollinator species may be able to colonize sites after some generations of introgressive hybridization via pollen (pollen swamping) and capture the haplotypes of the species previously growing there (Petit *et al.*, 2004). Such pollen swamping explains the presence of the two haplotypes of the Iberian lineage in the northwesternmost population of our study in the foothills of the Vosges Mountains (population 223, Sigolsheim) which might have arrived with another oak species from the west and have been captured by *Q. pubescens*. Despite interwoven recolonization pathways and, obviously, past introgression, the populations west of the previously mentioned genetic barrier in northern Italy all form a common genetic cluster when looking at nuclear microsatellites. This suggests that, even if *Q. pubescens* partly spread via pollen swamping, populations of this species in this area have a common origin. At the same time, it is remarkable that intermingling between the eastern and the western genetic cluster in northern Italy is rather limited, as supported by the Bayesian cluster and *Barrier* analysis.

The question is raised: why did *Q. pubescens* from the west and the east remain genetically differentiated despite a high number of generations since the onset of the Holocene which could have allowed exchange via long-distance pollen flow? An explanation for this finding could be the relatively limited distribution of *Q. pubescens* in the contact zone between the two clusters in northern Italy and historically limited gene flow between the two genetic clusters. Notably, *Q. pubescens* is absent from the Po plain, just south of the Italian Alps (Wellstein and Spada, 2015). Therefore, the occurrence of *Q. pubescens* is confined to a narrow zone along the Alpine foothills. Even along this corridor, deciduous oak forests with *Q. pubescens* are found interchanged with relict Mediterranean formations of the evergreen *Q. ilex* which are remnants of more widespread forests that dominated during the Atlantic period of the Holocene between 8000 and 4200 years ago (Barbero, 1978; Brullo and Guarino, 1998). Therefore, post-glacial contact and interbreeding between these two genetic clusters on the southern side of the Alps might have rather occurred late during the late Holocene (after the Atlantic period). The extent of gene flow between western and eastern refugial origins might have been limited (1) by landscape barriers, resulting in a narrow strip between the Alps and the Po Valley, (2) by the occurrence of other types of forests which have a relict character today, as well as (3) by other human land-uses in an area with early human settlement (Brullo and Guarino, 1998). Our results show that historical gene flow between the corresponding refugial origins was not sufficient to substantially blur the genetic differentiation between the clusters.

### Population genetic structure in the west fits a rugged topography with significant landscape barriers

Besides the significant genetic differentiation between western and eastern populations, we also observed genetic structure within each of the two clusters. In particular, populations in the west exhibited a more pronounced substructure than in the east, supported by a higher *F*_ST_, as well as by the Bayesian clustering and *Barrier* analyses. We interpret this to be the result of a rougher terrain in the area, but also of long-term isolation of some marginal populations with relict character. For instance, the separate cluster formed by the populations in Valais can be explained by its topographic position being surrounded by high mountains on three sides, strongly limiting gene flow. On the other hand, populations along the Jura Mountains in Switzerland appear to be genetically homogeneous, which can be attributed to the lack of significant landscape barriers along a southwest to northeast transect within this region. In the Upper Rhine lowlands, at the northern margin of the distribution, genetic differentiation is high despite the lack of landscape barriers. This agrees with previous results from this area (Neophytou *et al.*, 2015, 2024). Here, *Q. pubescens* mostly occurs in small and isolated populations, which are assumed to be relicts of larger and more widespread populations during the Atlantic climatic optimum (Leuschner and Ellenberg, 2017; Reif and Trué, 2023). Due to their small size following the climatic optimum (around 6200 years BP), these presumably relict populations might have experienced stronger genetic drift. At the same time, strong isolation among them might have limited gene flow.

Compared to their western counterparts, the populations assigned to the eastern genetic cluster are genetically more homogeneous. This might be explained by the fact that they are closer to one of the two centres of the species distribution which is located in southeastern Europe under a more pronounced continental climate (Wellstein and Spada, 2015; Leuschner and Ellenberg, 2017). Unlike central and southwestern parts of Central Europe (including most of the region covered by the western cluster), thermophilous oak forests dominated by *Q. pubescens* are important components of the zonal vegetation over large parts of the lowlands within and around the Pannonian Basin (Škvorc *et al.*, 2005; Čarni *et al.*, 2009; Fekete *et al.*, 2016), but also in Northeast Italy (Bolognini and Nimis, 1993). The eastern populations are located close or adjacent to this area, being situated at its northwestern margin. In addition, their haplotypic composition also agrees with the hypothesis of their origin from secondary (Younger Dryas) refugia in the southeast of the Alps and around the Pannonian Basin (see discussion above). Therefore, the weaker genetic structure and the limited fragmentation of their gene pool suggests that they are part of a larger and more homogeneous gene pool having its centre east-southeast of our study area. The fact that the genetic diversity was higher in the eastern compared to the western cluster is also supportive of this hypothesis.

Even if genetic differentiation was lower, genetic structure among populations of the eastern cluster was also present and is in concordance with geographical features in this region. For example, *Structure* analysis revealed a genetic differentiation of populations 202–205 from South Tyrol which can be explained by their occurrence in the Adige valley encircled by high mountains, but also admixture with the western cluster. Another group includes populations 201 and 202, both located in the Vienna Woods. The other two clusters are represented by the remaining populations in eastern Austria (206–208), as well as the two populations in Hungary (216–217).

### Introgression with Q. petraea seems to be more prevalent in the west

Genetic admixture between *Q. pubescens* and *Q. petraea* increased towards the west. In general, white oak species, especially *Q. petraea* and *Q. pubescens*, frequently produce fertile hybrids, due to weak reproductive barriers (Arnold, 1997; Lepais and Gerber, 2011; Lepais *et al.*, 2013). In particular, in natural populations north of the Alpine watershed, intermediate phenotypes are commonly found in sympatry with their parental taxa, suggesting extensive hybridization and introgression between these two species in this area (Müller, 1999; Neophytou *et al.*, 2015, 2024; Rellstab *et al.*, 2016*a*; Reutimann *et al.*, 2020). Based on the ecological niche and different climatic optima of the two species, introgression may also exhibit an adaptive advantage in certain environments (Reutimann *et al.*, 2023). On the one hand, *Q. pubescens* can tolerate summer heat and drought, along with a certain degree of winter cold, which explains its more widespread occurrence in southeastern Europe, where it grows under a warm but also increasingly continental climate. On the other hand, *Q. petraea* has its niche centre in areas with more moist and cooler site conditions and has been shown to be a generalist species that occurs in a wide range of ecological conditions (Rellstab *et al.*, 2016*b*). It is therefore more common in temperate climate areas of Europe with a more pronounced oceanic character (Leuschner and Ellenberg, 2017). In addition, *Q. pubescens* gradually loses its competitive ability at high elevations, while the natural range of *Q. petraea* includes submontane and montane sites (Reutimann *et al.*, 2023).

Consequently, we hypothesize that genetic admixture between the two species might serve as a means of exchanging genetic variation at loci coding for fitness-related traits (adaptive introgression). Leroy *et al.* (2020) postulated that the introgression of *Q. robur* alleles has promoted the expansion of *Q. petraea* populations to higher elevations in the Pyrenees. Similarly, introgression of alleles from *Q. petraea* into *Q. pubescens* and vice versa could increase the fitness of a species depending on site conditions. In our study, we found a higher level of admixture in sites at lower longitude, and with higher precipitation and higher moisture, and tend to be more north-exposed with higher temperature seasonality. On such sites, introgression of fitness-related alleles from *Q. petraea* could increase the adaptive capacity of *Q. pubescens*. Conversely, *Q. pubescens* could be a source for adaptive alleles for *Q. petraea* when the latter species is exposed to drier and warmer conditions (Reutimann *et al.*, 2023). The increase in the introgression index could also result from the more frequent occurrence of *Q. petraea* in or close to the studied populations, which is more associated with an oceanic climate (north and west of the study area). However, it is currently not possible to quantify the actual occurrence of individual white oak species across the study area.

Introgression from other species, especially from *Q. petraea*, could also be a reason for the high differentiation among some populations, particularly in the western part of our study area. Despite the taxonomic classification we undertook using *Structure*, it is possible that alleles introgressed from *Q. petraea* into *Q. pubescens* have inflated what we consider as intraspecific genetic (sub)structure. This might be the case for populations 243 (Valais) and 221 (Upper Rhine), which differ significantly from other closely situated populations of *Q. pubescens*, as revealed by both *Structure* and *Barrier* analyses, and which additionally display a high level of introgression. Thus, individuals assigned as pure but originating from hybrids several generations ago may still carry alleles introduced from *Q. petraea*. Varying intensities of past introgression may finally result in different frequencies of such alleles in these *Q. pubescens* populations. In accordance with this hypothesis, a study based on leaf morphometry revealed population-specific trichome types in the Upper Rhine and explained as the result of varying levels of introgression from *Q. petraea* (Höcke *et al.*, 2010). Finally, introgression from other oak species, mainly *Q. petraea*, can also explain the generally higher within-population genetic diversity of *Q. pubescens* in Central Europe which has been shown both for nuclear microsatellites shown in the present, but also in previous studies (Neophytou *et al.*, 2024) and agrees with results based on isozymes (Müller, 1999).

### Conclusions, implications for forest conservation and management

Our study provides valuable insights into the population genetic structure of *Q. pubescens* at the northern margin of its natural range as shaped by past demography. It also fills a gap in population genetic research of European white oaks. Analyses at a similar geographical scale have been conducted in Central Europe in *Q. petraea* and *Q. robur*, but have been rarer and more spatially restricted in *Q. pubescens*. We observed a structured gene pool of peri- and inner-Alpine *Q. pubescens* which is in line with the fact that this geographical area is located at the margin of the species’ distribution range. The two sister species, *Q. petraea* and *Q. robur*, appear to be genetically more homogeneous in Central Europe, likely due to the region’s central position in their natural distribution, in contrast to Q. pubescens (Neophytou *et al.*, 2024). Our results also contribute to a better understanding of the environmental conditions under which hybridization takes place, leading to locally high genetic admixture with *Q. petraea*. Also, the combination of nuclear and cpDNA analysis enables a more detailed assessment of the relationship between past migration and extant population genetic structure and diversity.

Looking to the future, this research can be relevant for conservation and seed sourcing strategies, such as delineating gene conservation units or regions of provenance for forest reproductive material (FRM). It would be of particular interest to address whether the subdivision of *Q. pubescens* into two large genetic clusters is of adaptive relevance. Further analyses combining, for example, environmental and genomic data may identify loci associated with local adaptation and reveal differing adaptation strategies within genetic clusters based on distinct genetic architectures.

Whereas large populations may be suitable both for *in situ* conservation and FRM harvesting (Bordács *et al.*, 2019), most of our study populations are small in size. In this case, e*x situ* conservation might be more suitable. Additionally, seed orchards may be a way to ensure a high number of reproducing trees and, thus, a high FRM diversity. The genetic structure described in this study could guide the geographical distribution of individuals selected both for establishing seed orchards and for *ex situ* conservation, for example in gene banks. Also, they can be used to inform decisions on population management, such as to enlarge populations via plantation. Under rapidly changing climatic conditions, it is important to ensure a high evolutionary potential of forest tree populations, i.e. a high capacity to adapt and persist under the new climatic conditions. To this end, hybridization and introgression between *Q. pubescens* and related species, especially *Q. petraea*, could serve as a resource to enhance adaptation to climate change. In particular, introgression from a more drought-tolerant species like *Q. pubescens* into a more demanding species like *Q. petraea* could improve the capacity of the latter’s ability to cope with climate change. On the other hand, past evolution has led to a genetic uniqueness of several of our *Q. pubescens* populations, which is partly reflected in the local morphological traits (Höcke *et al.*, 2010). Although hybridization may be beneficial in the context discussed above, emphasis should be placed on the conservation of such unique gene pools. Our study provides an important basis for identifying *Q. pubescens* populations which should be prioritized for conservation.

## SUPPLEMENTARY DATA

Supplementary data are available at *Annals of Botany* online and consist of the following.

Table S1: Overview of the analysed populations. Table S2: Nuclear microsatellite markers analysed in this study. Table S3: Chloroplast microsatellite markers used in this study. Table S4: Overview of *q*-values, introgression index and environmental variables of *Quercus pubescens* populations included in the generalized linear models. Table S5: Pairwise Pearson’s correlation coefficient among environmental variables. Table S6: Different modes for *K*-values, number of runs within each mode and average estimated ln probability of data [ln*P*(*X*|*K*)] and mean similarity scores (according to Jakobsson and Rosenberg, 2007) per mode for the *Structure* analysis with *Q. pubescens*, *Q. petraea* and *Q. robur*. Table S7: Number of individuals found for the different species. Table S8: Pairwise *F*_ST_ after Nei, used for the PCoA. Table S9: Different modes for *K*-values, number of runs within each mode and average estimated ln probability of data [ln*P*(*X*|*K*)] and mean similarity scores (according to Jakobsson and Rosenberg, 2007) per mode for the *Structure* analysis with all pure *Q. pubescens*. Table S10: Different modes for *K*-values, number of runs within each mode and average estimated ln probability of data [ln*P*(*X*|*K*)] and mean similarity scores (according to Jakobsson and Rosenberg, 2007) per mode for the *Structure* analysis with Q. pubescens from the western cluster. The selected K is shaded with grey. Table S11: Different modes for *K*-values, number of runs within each mode and average estimated ln probability of data [ln*P*(*X*|*K*)] and mean similarity scores (according to Jakobsson and Rosenberg, 2007) per mode for the *Structure* analysis with *Q. pubescens* from the eastern cluster. Table S12: Genetic diversity based on 16 nuclear microsatellite loci, Nind = number of individuals, Ho = observed heterozygosity ± standard error (s.e.), He = expected heterozygosity ± s.e., AR8 = allelic richness with rarefaction (rarefaction size = 8 individuals) ± s.e., FIS = inbreeding coefficient. Table S13: Chloroplast haplotypes identified with the eight cpSSRs, PCR-RFLP = the corresponding haplotypes and lineage (A, B, C) from Petit *et al.* (2002*a*). Table S14: Haplotypic diversity within population (hs) ± s.e. and genetic differentiation (GST) ± s.e. for all *Q. pubescens* populations and the subsets east and west using chloroplast microsatellites. Table S15: Summary of the final model after stepwise selection procedure.

Figure S1: Barplots with membership proportions of individuals and populations to one of *K* = 2–10 run clusters for the main modes for each *K* (to which most replicates with a *K* were assigned by *Clumpak*) for the interspecific *Structure* analysis. Figure S2: Mean estimated ln probability of data, ln*P*(*X|K*) and standard deviation (bars) averaged over 20 runs by number of assumed clusters (*K*) performed for each *K* (indicated with blue dots) for the *Structure* analysis with *Q. pubescens*, *Q. petraea* and *Q. robur*. Figure S3: Number of clusters to which at least one of our study populations was assigned based on the median (*MedMed K*, *MaxMed K*) or arithmetic mean of membership proportion (*MedMean K*, *MaxMean K*) for each *K* with a threshold of 0.5 according to Puechmaille (2016). Figure S4: Barplots with membership proportions of individuals and populations to one of *K* = 2–10 clusters for the minor modes (mode label to the right) for each *K* of the interspecific *Structure* analysis. Figure S5: Mean estimated ln probability of data, ln*P*(*X|K*), and standard deviation (bars) averaged over 20 runs by number of assumed clusters (*K*) performed for each *K* (indicated with blue dots) for the *Structure* analysis performed with all pure *Q. pubescens*. Figure S6: Barplots with membership proportions of individuals and populations to one of *K* = 2–10 run clusters for the main modes for each *K* (to which most replicates with a *K* were assigned by *Clumpak*) for the *Structure* analysis with all *Q. pubescens*. Figure S7: Number of clusters to which at least one of our study populations was assigned with *q* ≥ 0.5 based on the median (*MedMed K*, *MaxMed K*) or arithmetic mean of membership proportion (*MedMean K*, *MaxMean K*) for each consecutive *K* according to Puechmaille (2016). Figure S8: Barplots with membership proportions of individuals and populations to one of *K* = 2–10 run clusters for the minor modes (mode label to the right) for each *K* of the *Structure* analysis with all *Q. pubescens*. Figure S9: PCoA of only the pure *Q. pubescen*s (using *F*_ST_ values after Nei). Figure S10: Mean estimated ln probability of data, ln*P*(*X*|*K*), and standard deviation (bars) averaged over 20 runs by number of assumed clusters (*K*) performed for each *K* (indicated with blue dots) for the *Structure* analysis in *Q. pubescens* from the western cluster.. Figure S11: Barplots with membership proportions of individuals and populations to one of *K* = 2–10 run clusters for the main modes for each *K* (to which most replicates with a *K* were assigned by *Clumpak*) for the *Structure* analysis with *Q. pubescens* from the west. Figure S12: Number of clusters to which at least one of our study populations was assigned with *q* ≥ 0.5 based on the median (*MedMed K*, *MaxMed K*) or arithmetic mean of membership proportion (*MedMean K*, *MaxMean K*) for each consecutive *K* according to Puechmaille (2016). Figure S13: Barplots with membership proportions of individuals and populations to one of *K* = 2–10 run clusters for the minor modes (mode label to the right) for each *K* of the *Structure* analysis with *Q. pubescens* from the west.. Figure S14: Mean estimated ln probability of data, ln*P*(*X*|*K*), and standard deviation (bars) averaged over 20 runs by number of assumed clusters (*K*) performed for each *K* (indicated with blue dots) for the *Structure* analysis in *Q. pubescens* from the eastern cluster. Figure S15: Barplots with membership proportions of individuals and populations to one of *K* = 2–10 run clusters for the main modes for each *K* (to which most replicates with a *K* were assigned by *Clumpak*) for the *Structure* analysis with *Q. pubescens* from the east. Figure S16: Number of clusters to which at least one of our study populations was assigned with *q* ≥ 0.5 based on the median (*MedMed K*, *MaxMed K*) or arithmetic mean of membership proportion (*MedMean K*, *MaxMean K*) for each consecutive *K* according to Puechmaille (2016). Figure S17: Barplots with membership proportions of individuals and populations to one of *K* = 2–10 run clusters for the minor modes (mode label to the right) for each *K* of the *Structure* analysis with *Q. pubescens* from the east.

mcae216_suppl_Supplementary_Tables_S1-S15

mcae216_suppl_Supplementary_Figures_S1-S17

## Data Availability

Genotypic data used for the analysis are available at the Dryad digital repository, https://doi.org/10.5061/dryad.vhhmgqp4d.
