# Supplementary material for: The influence of post-glacial migration and hybridization on the gene pool of marginal Quercus pubescens populations in Central Europe
Source: Ann Bot. 2024 Dec 19;135(5):867–84. doi: 10.1093/aob/mcae216 (PMC12064428; doi:10.1093/aob/mcae216)
Supplement: mcae216_suppl_Supplementary_Figures_S1-S17 [file mcae216_suppl_supplementary_figures_s1-s17.docx]

**Supplementary material**

Figures S1 – S1

**
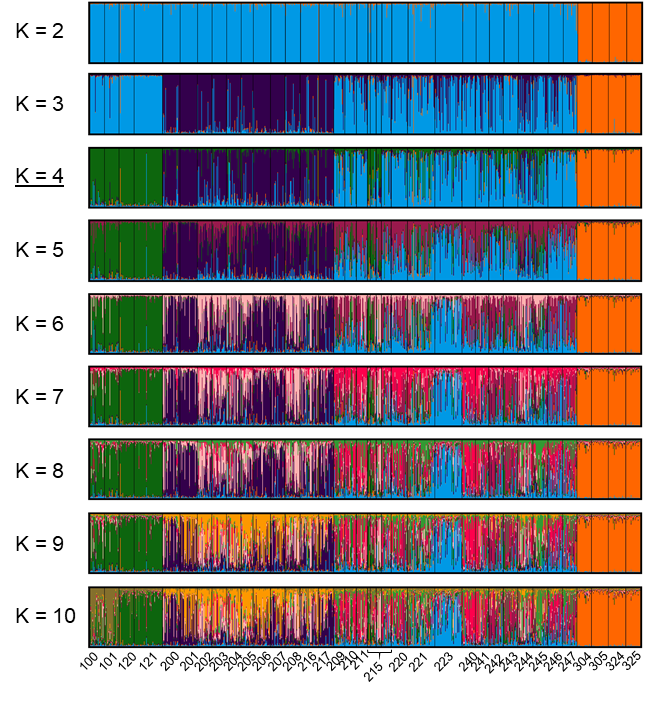
**

**Figure S1** – Barplots with membership proportions of individuals and populations to one of K = 2 to 10 run clusters for the main modes for each K (to which most replicates with a K were assigned by *Clumpak*) for the interspecific *Structure* analysis. 100, 101, 120, 121 = *Quercus petraea*, 304, 305, 324,325 = *Q. robur*, all others = *Q. pubescens* (designated). The K for the selected clustering solutiounderscored.is underscored.324,325 = *Q. robur*, all others = *Q. pubescens* (designated). The K for the selected clustering solution is underscored.


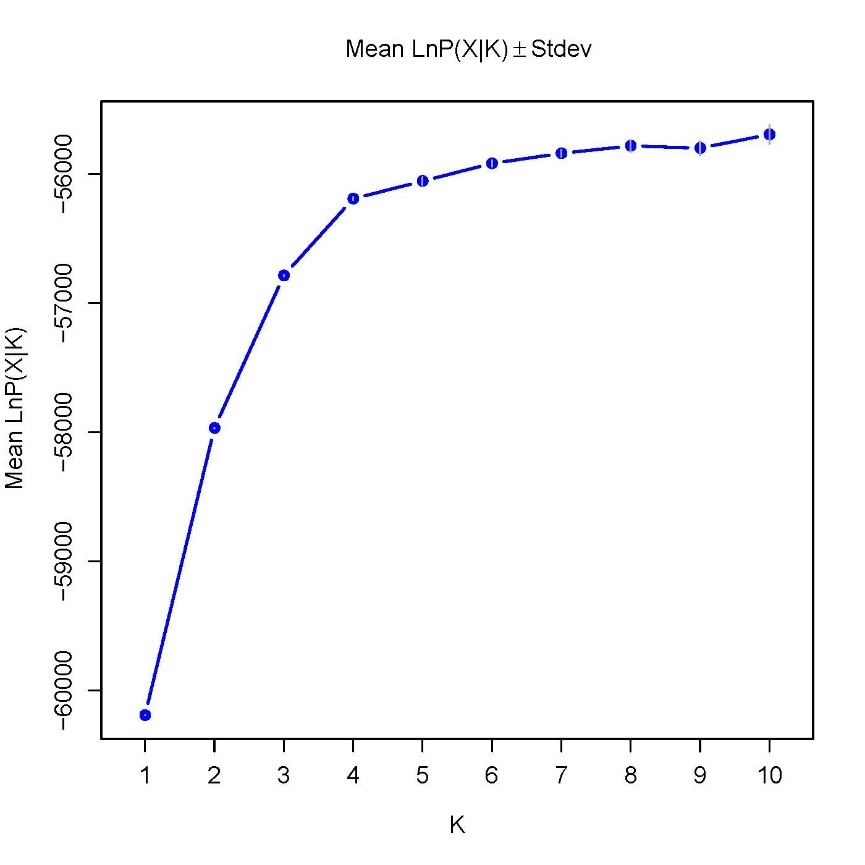

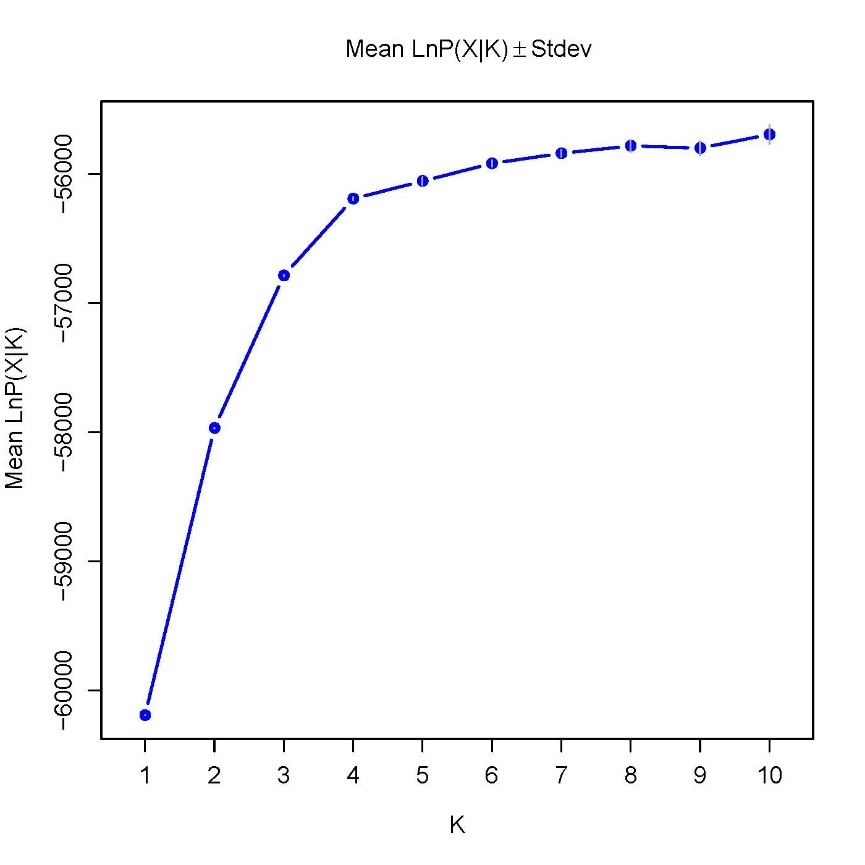


**Figure S2** – Mean Estimated ln probability of data, *lnP(X|K)* and standard deviation (bars) averaged over 20 runs by number of assumed clusters (*K*) performed for each *K* (indicated with blue dots) for the Structure analysis with *Q. pubescens*, *Q. petraea* and *Q. robur*.


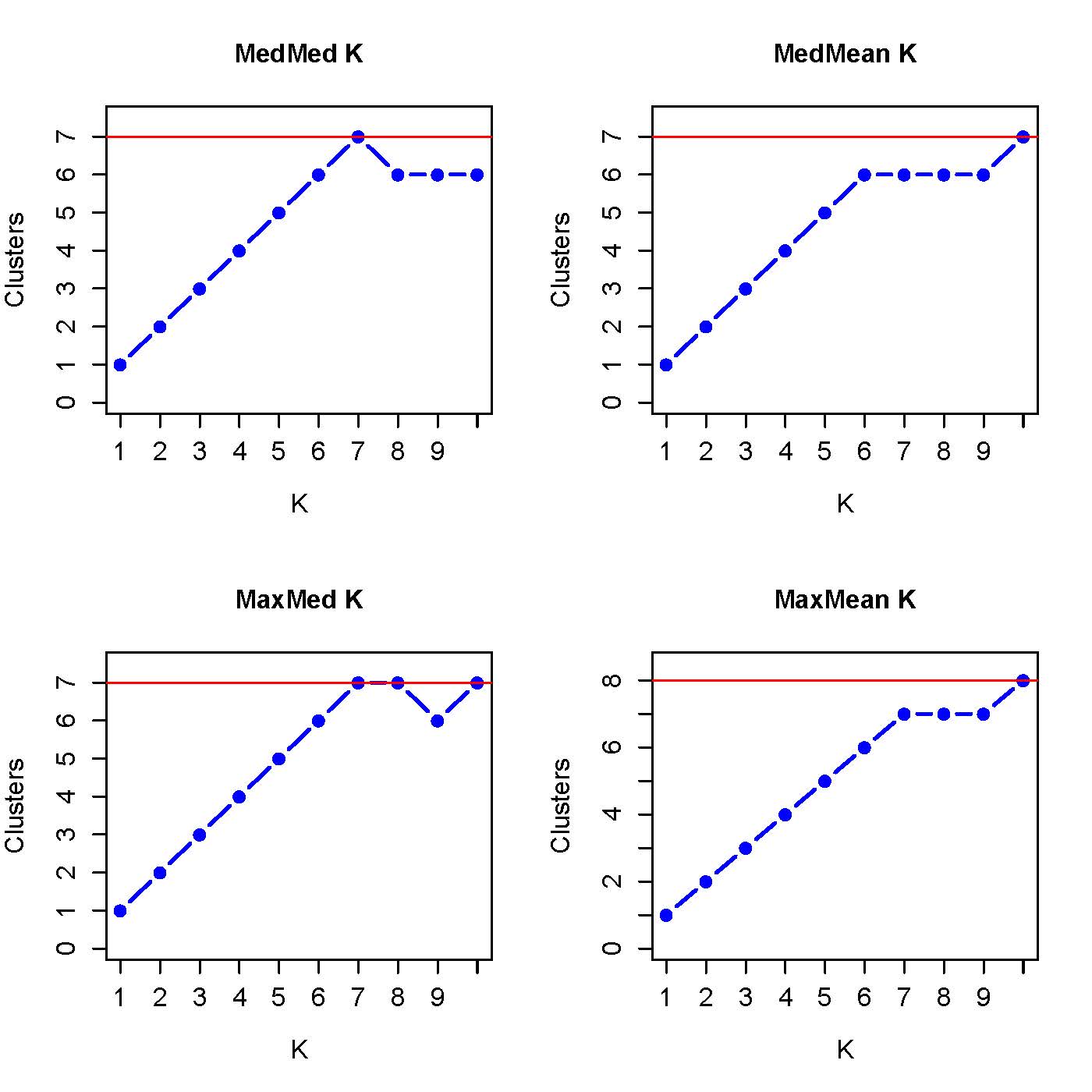


**Figure S3** – Number of clusters to which at least one of our study populations was assigned based on the median (*MedMed K*, *MaxMed K*) or arithmetic mean of membership proportion (*MedMean K*, *MaxMean K*) for each opted K with a threshold of 0,5 according to Puechmaille (2016). The metrics *MedMed K* and *MedMean K* express this number as the median across the 20 replicate runs performed per K. *MaxMed K* and *MaxMean K* express this number as the maximum across these runs within K. Results for the interspecific Structure analysis.


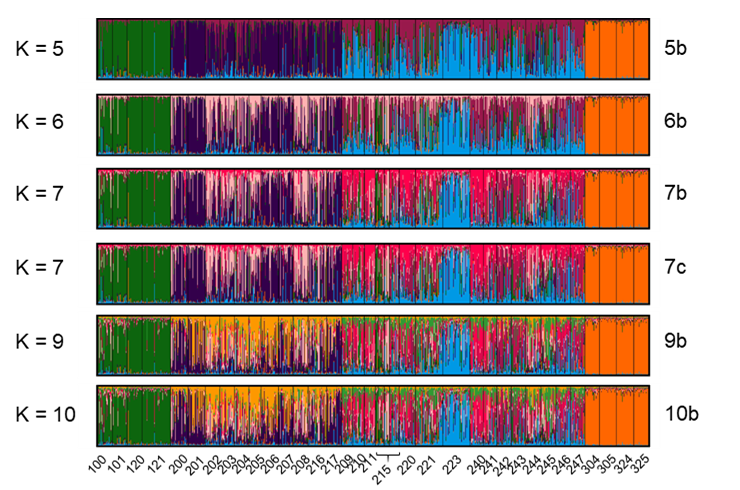


**Figure S4** – Barplots with membership proportions of individuals and populations to one of K = 2 to 10 clusters for the minor modes (mode label to the right) for each K of the interspecific Structure analysis. 100, 101, 120, 121 = *Quercus petraea*, 304, 305, 324, 325 = *Q. robur,* all others = *Q. pubescens* (designated).


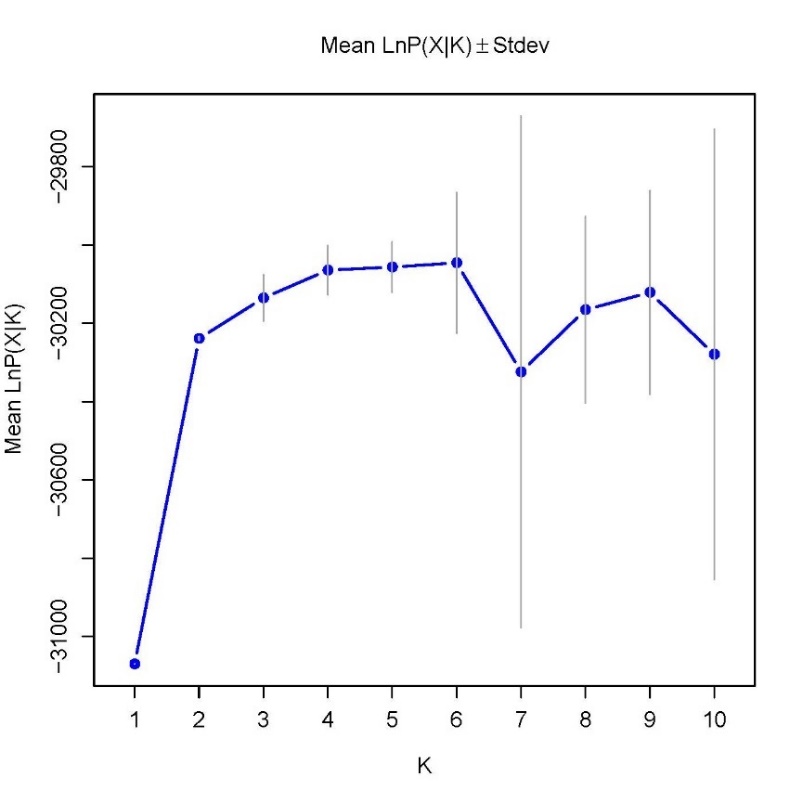


**Figure S5** – Mean Estimated ln probability of data, *lnP(X|K)*, and standard deviation (bars) averaged over 20 runs by number of assumed clusters (*K*) performed for each *K* (indicated with blue dots) for the *Structure* analysis performed with all pure *Q. pubescens*.


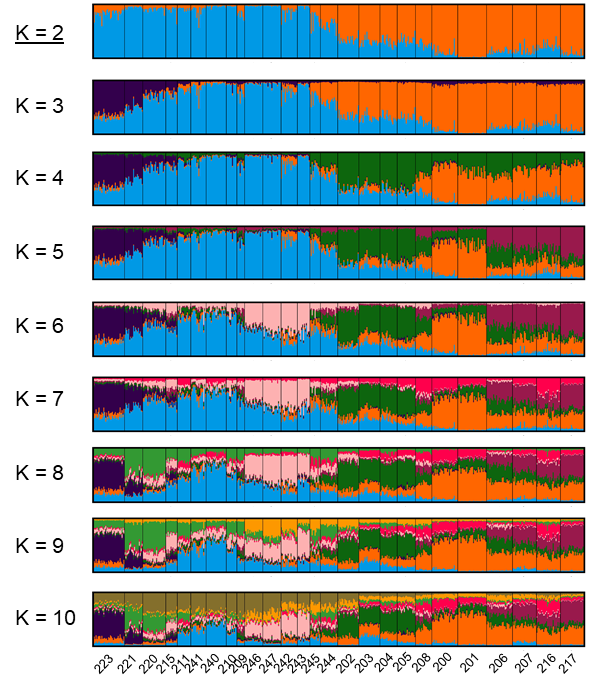


**Figure S6** – Barplots with membership proportions of individuals and populations to one of K = 2 to 10 run clusters for the main modes for each K (to which most replicates with a K were assigned by *Clumpak*) for the *Structure* analysis with all *Q. pubescens*. The K for the selected clustering solution is underscored.


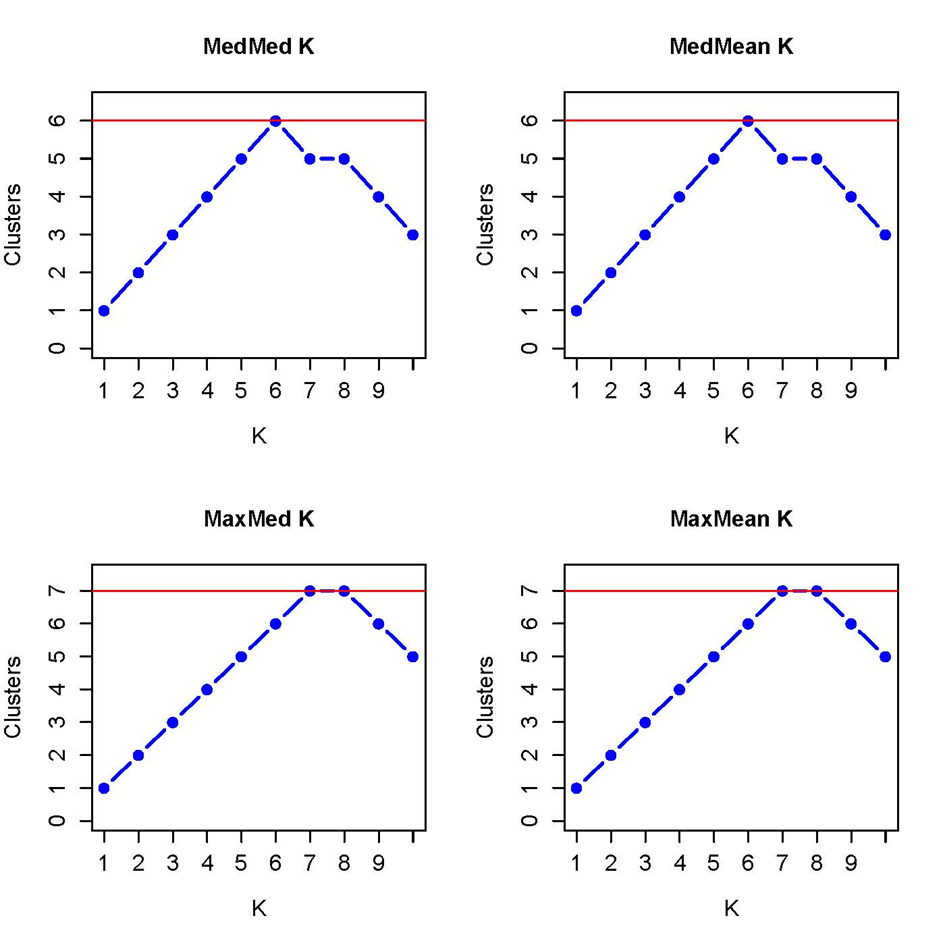


**Figure S7** – Number of clusters to which at least one of our study populations was assigned with q ≥ 0.5 based on the median (*MedMed K*, *MaxMed K*) or arithmetic mean of membership proportion (*MedMean K*, *MaxMean K*) for each consecutive K according to Puechmaille (2016). The metrics *MedMed K* and *MedMean K* express this number as the median across the 20 replicate runs performed per K. *MaxMed K* and *MaxMean K* express this number as the maximum across these runs within K. Results for the *Structure* analysis including all *Q. pubescens*.


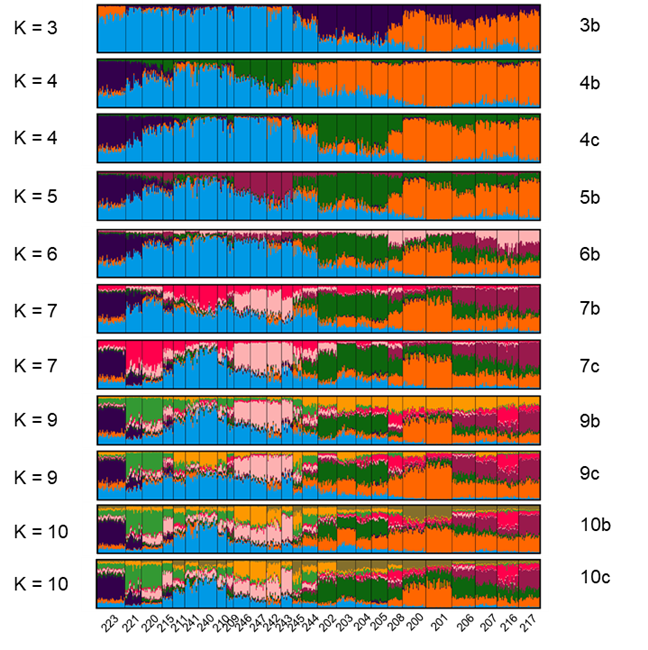


**Figure S8** - Barplots with membership proportions of individuals and populations to one of K = 2 to 10 run clusters for the minor modes (mode label to the right) for each K of the Structure analysis with all *Q. pubescens*.


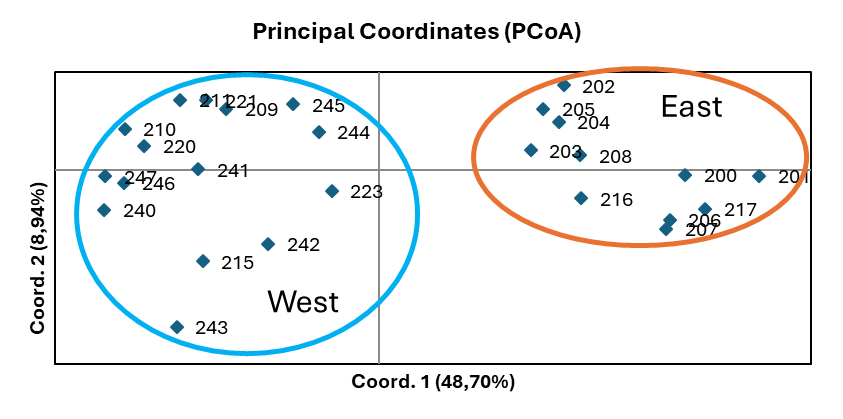


**Figure S9** – PCoA of only the pure *Q. pubescen*s (using F_ST_-values after Nei)


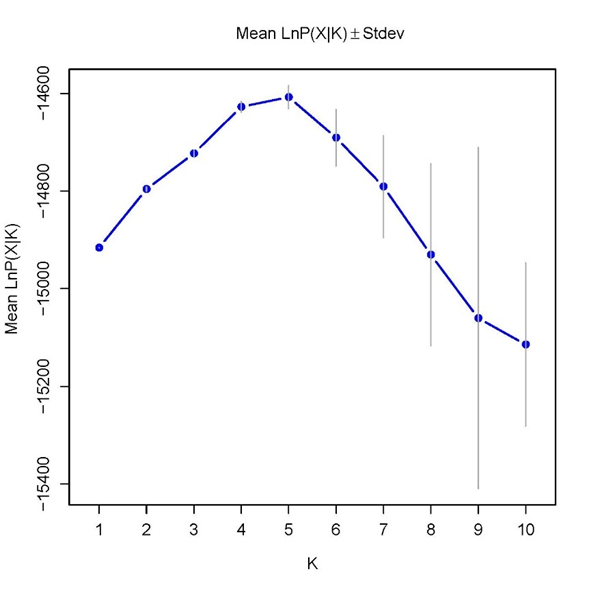


**Figure S10** – Mean Estimated ln probability of data, lnP(X|K), and standard deviation (bars) averaged over 20 runs by number of assumed clusters (K) performed for each K (indicated with blue dots) for the Structure analysis in *Q. pubescens* from the western cluster.

|  |  |  |  |  |
| --- | --- | --- | --- | --- |


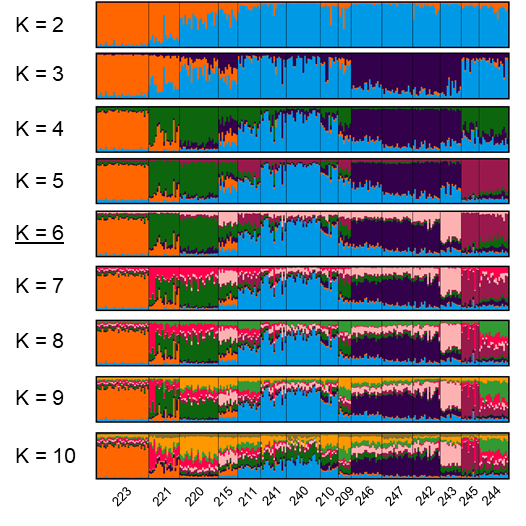


**Figure S11** – Barplots with membership proportions of individuals and populations to one of K = 2 to 10 run clusters for the main modes for each K (to which most replicates with a K were assigned by *Clumpak*) for the *Structure* analysis with *Q. pubescens* from the west. The K for the selected clustering solution is underscored.


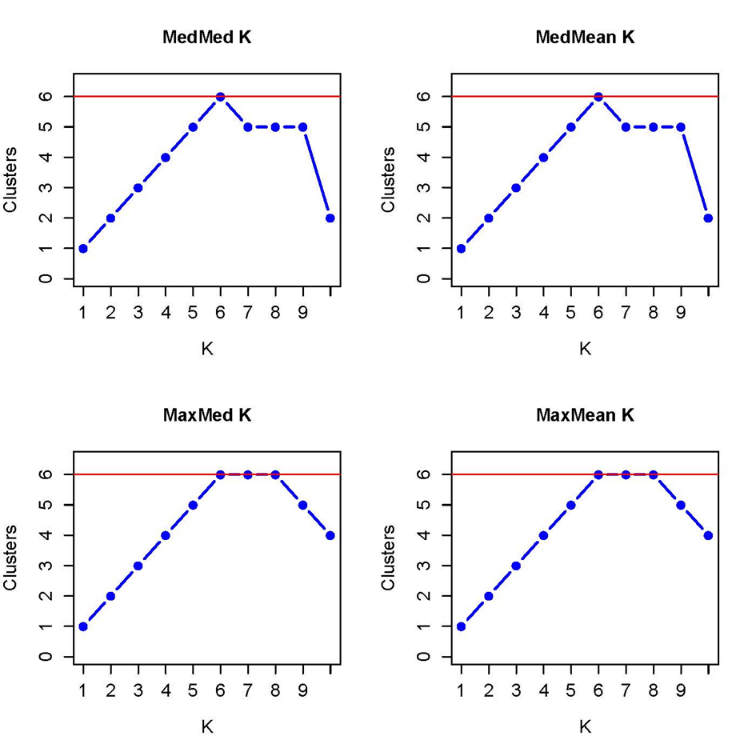


**Figure S12** – Number of clusters to which at least one of our study populations was assigned with q ≥ 0.5 based on the median (*MedMed K*, *MaxMed K*) or arithmetic mean of membership proportion (*MedMean K*, *MaxMean K*) for each consecutive K according to Puechmaille (2016). The metrics *MedMed K* and *MedMean K* express this number as the median across the 20 replicate runs performed per K. *MaxMed K* and *MaxMean K* express this number as the maximum across these runs within K. Results for the run with western populations of *Q. pubescens*.


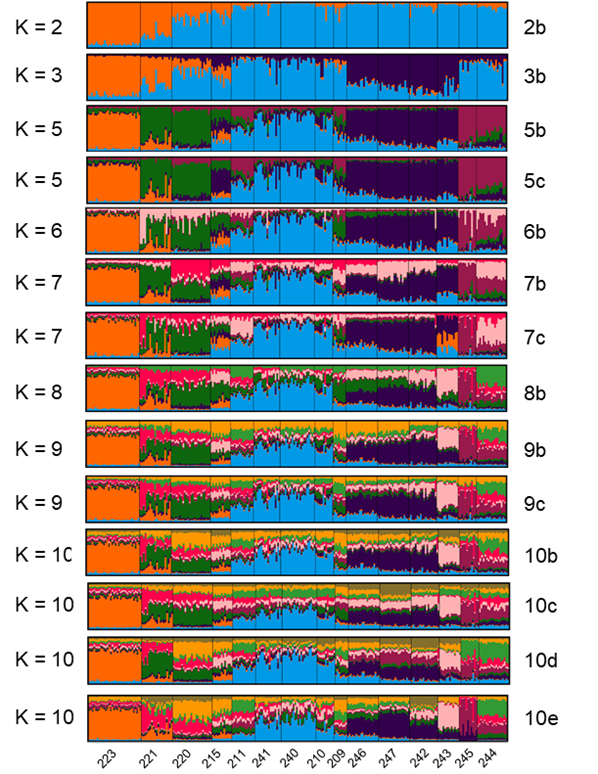


**Figure S13** – Barplots with membership proportions of individuals and populations to one of K = 2 to 10 run clusters for the minor modes (mode label to the right) for each K of the Structure analysis with *Q. pubescens* from the west.


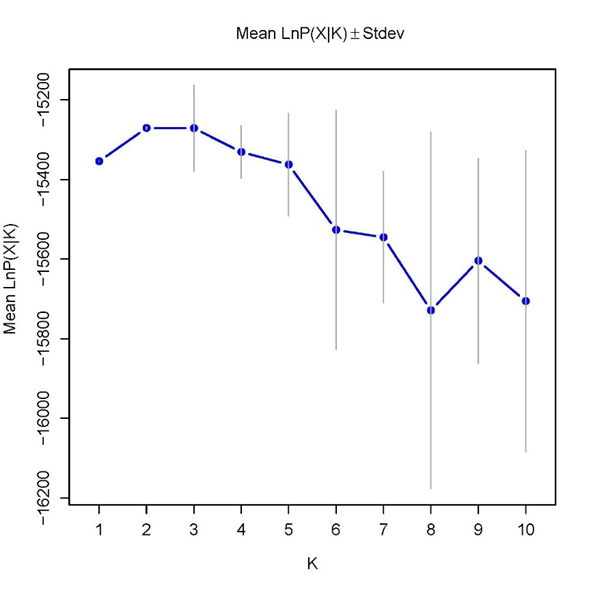


**Figure S14** – Mean Estimated ln probability of data, lnP(X|K), and standard deviation (bars) averaged over 20 runs by number of assumed clusters (K) performed for each K (indicated with blue dots) for the Structure analysis in *Q. pubescens* from the eastern cluster.


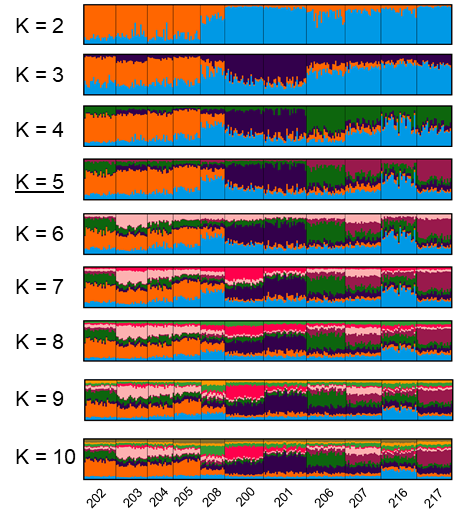


**Figure S15** – Barplots with membership proportions of individuals and populations to one of K = 2 to 10 run clusters for the main modes for each K (to which most replicates with a K were assigned by *Clumpak*) for the *Structure* analysis with *Q. pubescens* from the east. The K for the selected clustering solution is underscored.


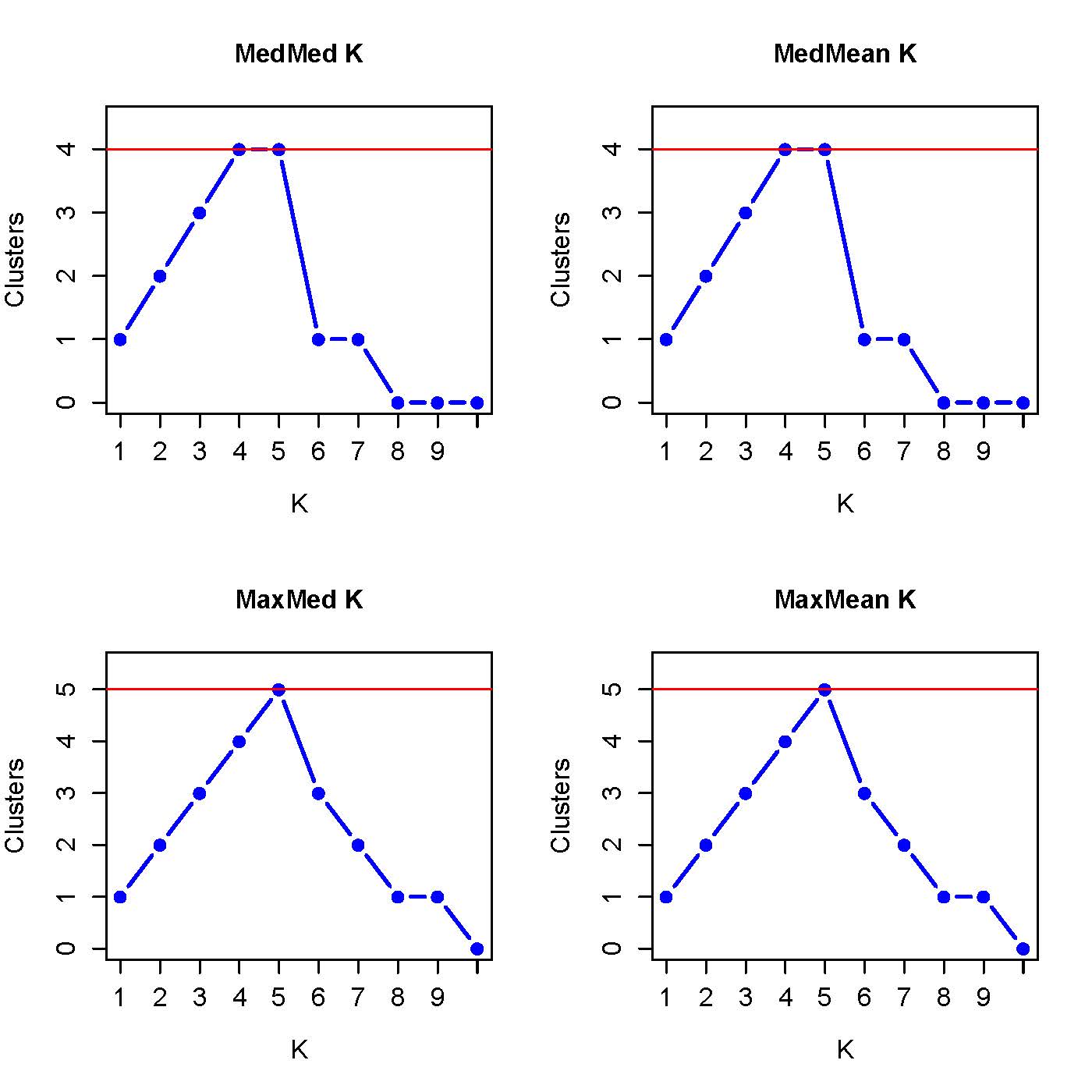


**Figure S16** – Number of clusters to which at least one of our study populations was assigned with q ≥ 0.5 based on the median (*MedMed K*, *MaxMed K*) or arithmetic mean of membership proportion (*MedMean K*, *MaxMean K*) for each consecutive K according to Puechmaille (2016). The metrics *MedMed K* and *MedMean K* express this number as the median across the 20 replicate runs performed per K. *MaxMed K* and *MaxMean K* express this number as the maximum across these runs within K. Results for the run with eastern populations of *Q. pubescens*.


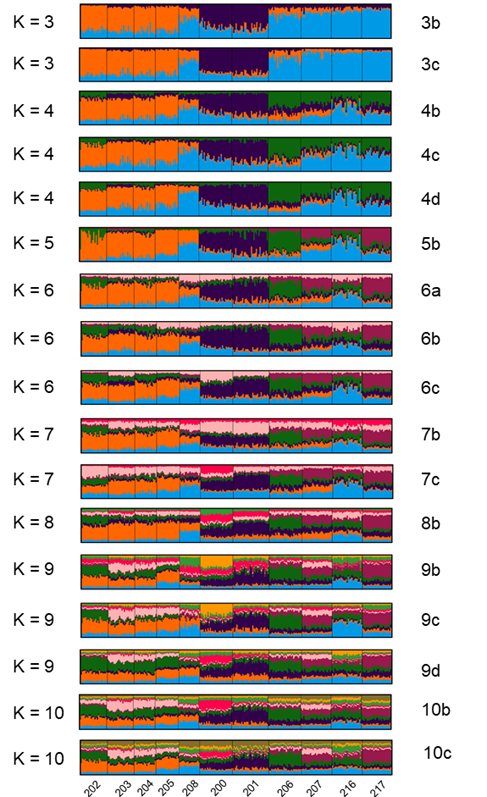


**Figure S17** – Barplots with membership proportions of individuals and populations to one of K = 2 to 10 run clusters for the minor modes (mode label to the right) for each K of the Structure analysis with *Q. pubescens* from the east.
